# Evaluation of the Cochrane Collaboration’s tool for assessing the risk of bias in randomized trials: focus groups, online survey, proposed recommendations and their implementation

**DOI:** 10.1186/2046-4053-3-37

**Published:** 2014-04-15

**Authors:** Jelena Savović, Laura Weeks, Jonathan AC Sterne, Lucy Turner, Douglas G Altman, David Moher, Julian PT Higgins

**Affiliations:** 1 School of Social and Community Medicine, University of Bristol, Bristol, UK; 2 Ottawa Integrative Cancer Centre, Ottawa, ON, Canada; 3 Ottawa Hospital Research Institute, Ottawa, ON, Canada; 4 Centre for Statistics in Medicine, University of Oxford, Oxford, UK; 5 Department of Epidemiology and Community Medicine, Faculty of Medicine, University of Ottawa, Ottawa, ON, Canada; 6 Centre for Reviews and Dissemination, University of York, York, UK

**Keywords:** Survey, Focus groups, Bias assessment, Quality assessment, Systematic reviews

## Abstract

**Background:**

In 2008, the Cochrane Collaboration introduced a tool for assessing the risk of bias in clinical trials included in Cochrane reviews. The risk of bias (RoB) tool is based on narrative descriptions of evidence-based methodological features known to increase the risk of bias in trials.

**Methods:**

To assess the usability of this tool, we conducted an evaluation by means of focus groups, online surveys and a face-to-face meeting. We obtained feedback from a range of stakeholders within The Cochrane Collaboration regarding their experiences with, and perceptions of, the RoB tool and associated guidance materials. We then assessed this feedback in a face-to-face meeting of experts and stakeholders and made recommendations for improvements and further developments of the RoB tool.

**Results:**

The survey attracted 380 responses. Respondents reported taking an average of between 10 and 60 minutes per study to complete their RoB assessments, which 83% deemed acceptable. Most respondents (87% of authors and 95% of editorial staff) thought RoB assessments were an improvement over past approaches to trial quality assessment. Most authors liked the standardized approach (81%) and the ability to provide quotes to support judgements (74%). A third of participants disliked the increased workload and found the wording describing RoB judgements confusing. The RoB domains reported to be the most difficult to assess were incomplete outcome data and selective reporting of outcomes. Authors expressed the need for more guidance on how to incorporate RoB assessments into meta-analyses and review conclusions. Based on this evaluation, recommendations were made for improvements to the RoB tool and the associated guidance. The implementation of these recommendations is currently underway.

**Conclusions:**

Overall, respondents identified positive experiences and perceptions of the RoB tool. Revisions of the tool and associated guidance made in response to this evaluation, and improved provision of training, may improve implementation.

## Background

Systematic reviews of randomized trials provide the best evidence about the effects of healthcare interventions. Nevertheless, randomized trials are not immune from bias. There is good empirical evidence [1-3] that flaws in particular aspects of trial conduct may lead to biased intervention effect estimates, which will then bias results of systematic reviews that aim to collate and synthesize all studies meeting pre-specified eligibility criteria. It is therefore important, in order to minimize bias in the conclusions of a systematic review, to consider potential limitations of each eligible study.

Systematic reviews produced by The Cochrane Collaboration have previously used a variety of methods to assess methodological quality of included trials [4]. There was no consistency between approaches recommended by different Cochrane Review Groups, most of the approaches were not evidence-based and many review groups used methods based on numerical scores, which have been shown to be inadequate [4,5]. In 2005, The Cochrane Collaboration’s Methods Groups initiated the development of a new strategy for addressing the quality of randomized trials. This project commenced with a 3-day meeting of statisticians, epidemiologists and review authors, held in Cambridge, UK, following which designated pairs of individuals wrote the first draft of different components of the tool. In brief, the Cochrane risk of bias (RoB) tool involves assessment of the risk of bias arising from each of six domains (generation of the allocation sequence, concealment of the allocation sequence, blinding, incomplete outcome data, selective outcome reporting and other biases). In contrast to previous approaches, this tool elicited judgements for the domain-level risk of bias, supported by narrative explanation of evidence-based methodological features known to increase the risk of bias in trials. The narrative description can include quotes from the papers that authors have used to inform their judgements. Another novel feature of the tool was that figures can be generated to display the RoB judgements graphically across included studies. The original version of the RoB tool (Table 1) was first published in 2008 in the *Cochrane Handbook for Systematic Reviews of Interventions*[6] and implemented in the Collaboration’s review-writing software, RevMan [7]. An updated version was published in 2011 [8,9].

**Table 1:** The original risk of bias tool

**Domain**	**Description**	**Review authors’ judgement**
Sequence generation	Describe the method used to generate the allocation sequence in sufficient detail to allow an assessment of whether it should produce comparable groups	Was the allocation sequence adequately generated?
Allocation concealment	Describe the method used to conceal the allocation sequence in sufficient detail to determine whether intervention allocations could have been foreseen in advance of, or during, enrolment	Was allocation adequately concealed?
Blinding of participants, personnel and outcome assessors. Assessments should be made for each main outcome (or class of outcomes)	Describe all measures used, if any, to blind study participants and personnel from knowledge of which intervention a participant received. Provide any information relating to whether the intended blinding was effective	Was knowledge of the allocated intervention adequately prevented during the study?
Incomplete outcome data. Assessments should be made for each main outcome (or class of outcomes)	Describe the completeness of outcome data for each main outcome, including attrition and exclusions from the analysis. State whether attrition and exclusions were reported, the numbers in each intervention group (compared with total randomized participants), reasons for attrition/exclusions where reported, and any re-inclusions in analyses performed by the review authors	Were incomplete outcome data adequately addressed?
Selective outcome reporting	State how the possibility of selective outcome reporting was examined by the review authors, and what was found	Are reports of the study free of suggestion of selective outcome reporting?
Other sources of bias	State any important concerns about bias not addressed in the other domains in the tool	Was the study apparently free of other problems that could put it at a high risk of bias?
	If particular questions/entries were pre-specified in the review’s protocol, responses should be provided for each question/entry	

Based on Higgins and Altman [6]. The original tool was in use at the time focus groups and survey were administered (September 2009 to February 2010).

In this paper, we describe the results of an evaluation of the initial version of The Cochrane Collaboration’s RoB tool following its launch in 2008, the resulting recommendations for amendments and current progress in their implementation. Objectives of the evaluation were to: 1) assess the usability of the tool; 2) assess the acceptability of the resources needed to use the tool; 3) identify areas authors are finding difficult to implement; and 4) identify additional training requirements.

## Methods

The evaluation of the RoB tool was initiated in early 2009. A planning meeting, comprising the organizing committee and other Cochrane contributors with relevant expertise and/or experience, including editors and other editorial office staff, was held during the 17th annual Cochrane Colloquium in Singapore in October 2009. The evaluation consisted of three stages.

First, a series of focus groups was held with a main goal of guiding the development of a questionnaire that would be subsequently used to survey stakeholders within The Cochrane Collaboration. Participants were invited to take part in focus groups via emails sent to a Cochrane Collaboration mailing list (CC-Info) and the focus groups were also listed in the program on the 17th Cochrane Colloquium website. Four 90-minute focus groups were held: one via teleconference and three in person during the Colloquium. The discussions were semi-structured and open-ended and were facilitated by one team member (DM, JACS, JS or LW). Questions focused on experiences with the RoB tool, perceptions about the level of difficulty in using the tool and in summarizing RoB assessments at different levels, confidence in RoB assessments and perspectives regarding the sufficiency and adequacy of available training materials, or reasons for non-use of the tool. The discussions were recorded and transcribed. Transcripts were coded using basic content analysis to identify questionnaire items and appropriate response categories.

Analysis of transcripts from the focus groups, together with the expertise of investigators and project staff, guided the development of three online questionnaires aimed at: 1) review authors who had used the tool; 2) review authors who had not used the tool (to ask about barriers); and 3) editorial teams within the Collaboration. Questionnaires were pilot tested before the survey was launched. Review authors who had used the RoB tool were asked questions assessing their experience of using the tool, including workload, opinions and perceptions of the tool, experience with specific bias domains, and training preferences (32 questions). Review authors who had not used the RoB tool were asked about reasons for not using the tool and about training preferences (nine questions). Review group staff were asked about their experiences of providing support to review authors (29 questions). Participants were recruited through established Cochrane Collaboration mailing lists. Links to each questionnaire were emailed to lists of review authors (5,038 subscribers), coordinating editors (79 subscribers), managing editors (69 subscribers) and to the general purpose email list, CC-Info (2,182 subscribers). The survey took place over a 3-week period in February 2010. The extent of subscriber overlap between these lists was unknown as they are maintained by different groups and are confidential. In addition, it was not possible to estimate the proportion of out-of-date or inactive subscribers in each list. Responses were analyzed using descriptive statistics, and free-text answers were analyzed by basic content analysis.

A face-to-face meeting was held in Cardiff, UK, in March 2010 to discuss results from the focus groups and surveys, and consider revisions to the first version of the RoB tool. There were 23 participants, including statisticians, epidemiologists, Cochrane review authors, editors and other members of Cochrane Review Groups and Cochrane Methods Groups, and the Editor in Chief of The Cochrane Library (http://www.thecochranelibrary.com). At the meeting, results from the focus groups and surveys were presented to initiate a semi-structured, open-ended discussion regarding specific aspects of implementation, while encouraging participants to raise issues they considered important. The discussion was guided by a set of topic areas identified as important through the survey. Recommendations for changes to the RoB tool and related guidance in the *Cochrane Handbook* were discussed and agreed through informal consensus.

In the months after the meeting, we collaborated with relevant groups within The Cochrane Collaboration to implement the proposed changes, including working with the software developers to integrate the proposed changes into Cochrane software and making arrangements for revising relevant guidance. As a part of a wider consultation within The Cochrane Collaboration about the proposed changes, an interactive discussion workshop was held at the 18th Cochrane Colloquium in Keystone, CO, USA. This was open to any Colloquium participants interested in attending. We presented the results from the online surveys as well as the proposed recommendations and invited participants to discuss the recommendations and provide feedback. Discussion points and feedback were recorded and fed back to the evaluation team and other groups within the Collaboration involved in the implementation of the recommendations. The implementation of proposed longer-term changes is ongoing and working groups were set up with the aim of continuous evaluation and development of the RoB tool.

This project was approved by the Ottawa Hospital Research Institute Ethics Committee (ON, Canada). The University of Bristol Faculty of Medicine and Dentistry Ethics Committee (Bristol, UK) classified this project as an audit of research practices, rather than a research project, and thus advised that explicit ethics approval was not required.

## Results

### Focus groups

The four focus groups involved 25 participants, the majority of whom were experienced users of the RoB tool. Others were familiar with the RoB tool but had not yet used it in the context of a Cochrane review. The main topics of discussion were: how the RoB tool is used in practice (for example pilot testing, updated reviews, modifications, use of quotes); opinions of the RoB tool (for example comparison to past practice, aspects liked and not liked); opinions of, and experiences with, specific domains; and current and desired training materials.

Focus group participants felt that the RoB tool was an improvement over past practice. Specific benefits described included: having a standardized approach to bias assessments; the transparency provided by requesting quotes; the flexibility of the tool; the figures that can be produced in RevMan (the Cochrane Collaboration’s software for systematic reviews and meta-analyses); providing a good framework for consideration of the risk of bias; and providing a platform to encourage critical thinking. Questions about these potential benefits were therefore included in the survey. The main drawbacks described, which were also addressed in the survey, included: the increased workload and complexity as compared with past practice; the subjectivity of assessments; and a lack of clarity regarding the meaning of the ‘Yes’, ‘No’ or ‘Unclear’ judgements. The original RoB tool phrased the judgements as answers to questions requiring a ‘Yes’, ‘No’ or ‘Unclear’ response, with ‘Yes’ reflecting a low risk of bias. Many participants deemed this wording to be confusing and instead expressed a preference for a direct response such as ‘Low risk’. The analysis of the focus group discussions identified important topics to cover in the survey and helped formulate survey questions and possible response options.

The focus groups also identified issues and suggestions that would require discussion during the subsequent face-to-face meeting relating to how the RoB tool is used in practice. For example, several participants raised the issue that RoB assessments present a particular problem when updating systematic reviews. Adopting the new tool in an updated review requires review authors to re-assess the risk of bias of studies included in the original review, which they were often unwilling to do, and Cochrane Review Groups were not resourced to do this on behalf of authors. Participants also suggested that graphical displays of RoB assessments across studies should be prepared separately for individual outcomes measured in the review rather than at the study level, as individual outcomes can be judged to be at higher or lower risk of bias using the tool. They further suggested that such figures should reflect the sizes of the studies rather than a simple count of how many studies were in each RoB judgement category, as had been implemented in RevMan.

Finally, training and guidance materials (for example the *Cochrane Handbook* guidance, workshops) were considered important by focus group participants. Most participants described these materials as clear, but editorial groups described a challenge in persuading authors to follow and understand the guidance. Participants also described a need for more, in particular online, training materials. A list of specific gaps in existing guidance was developed to guide future training needs. These include guidance on: how to use RoB assessments within systematic reviews; how to assess risk of bias for study designs other than randomized trials; and whether and when it might be appropriate to add specific items (for example reporting of power calculations, funding source) to the ‘other’ bias domain. For detailed focus group findings see Additional file 1: Appendix 4.

### Survey

In total, 380 respondents completed the survey. This represents a 4.4% response rate under assumptions that all subscribers’ emails were active and up-to-date, and that there was no overlap in subscribers between mailing lists. We received 190 responses from authors who had used the RoB tool and 132 from authors who had not (non-users). Of the 58 Cochrane Review Group staff who responded, 19 were managing editors, 11 coordinating editors, 11 editors and 17 other staff.

Non-users of the RoB tool were asked nine questions covering: reasons for not using the RoB tool; training needs; and opinions on the availability of training. Most non-user respondents identified themselves as likely future users, for example because: they had not conducted a Cochrane review since the introduction of the RoB tool (95 of the 132 respondents); their review was still in the protocol stage (four respondents); they had not yet started the RoB assessments for their review (three respondents); or their co-authors were tasked with completing the RoB assessments (four respondents). Only eight respondents stated that they preferred using another assessment method, and two stated that their reason for non-use of the tool was the time it would take to use it. The answers of non-user respondents to training-related questions are summarized in Table 2 and provided in detail in Additional file 1: Appendix 2.

**Table 2:** Extract of results from survey of Cochrane authors and Review Group staff: questions about training in risk of bias

**Survey questions**	**Authors (%)**		**Editorial staff (%) (n = 58)**
	**Users of RoB tool (n = 190)**	**Non-users (n = 132)**	
Training in RoB assessment (Q25, Q2, Q24)^a^			
Attended workshop at Cochrane Colloquium	74 (39)	14 (11)	29 (50)
Attended standard Cochrane author training	44 (23)	15 (11)	6 (10)
Read relevant material in own time	124 (65)	34 (26)	37 (64)
No specific training	29 (15)	84 (64)	9 (16)
Read guidance in *Cochrane Handbook* related to RoB tool (Q26, Q3, Q25)	178 (94)	44 (34)	55 (95)
Read Chapter 8 of the *Cochrane Handbook* (Q26a, Q3a, Q25a)^a^	144	27	44
Read *Cochrane Handbook* (Part 2) from start to finish	30	14	16
Used *Cochrane Handbook* to look up specific issues	147	27	47
Level of detail provided in the *Cochrane Handbook* is appropriate (Q26b, Q3b, Q25b)	133	39	42
Provision of additional examples would be beneficial (Q26c, Q3c, Q25c)	142	39	48
Received guidance from CRG related to RoB tool (Q27, Q4)	80 (42)	20 (16)	Not applicable
CRG provides guidance related to RoB tool to their authors (Q26)	Not applicable	Not applicable	44 (80)
Advice to read Chapter 8 of *Cochrane Handbook*^a^	43	8	41
Specific written advice developed by the CRG^a^	31	1	18
Specific verbal advice given by the CRG^a^	33	6	13
Advice provided by CRG rated good, very good or excellent (Q27b, Q4b)	67	19	Not asked
Availability of written guidance is sufficient (Q28, Q5, Q27)	139 (75)	75 (65)	36 (63)
Availability of training events is sufficient (Q29, Q6, Q28)	117 (68)	55 (50)	28 (50)
Format of training most likely to access (Q30, Q7)			Not asked
Training that is part of standard author training	32 (17)	18 (14)	
Online training, including webinars	102 (55)	74 (59)	
In-person workshops	51 (27)	28 (22)	
Level of training most likely to access (Q31, Q8)			Not asked
Beginning	22 (12)	60 (48)	
Intermediate	78 (41)	45 (36)	
Advanced	89 (47)	19 (15)	

Only the most frequent responses shown in the table, and some response options have been grouped to fewer categories. Not all respondents answered each question. Question numbers refer to the survey questions in Additional file 1: Appendices 1, 2 and 3, respectively. For full details of questions and responses, see Additional file 1: Appendices 1 to 4. ^a^ Respondents were allowed to select multiple answers for this question. *CRG*, Cochrane Review Group; *RoB*, risk of bias.

#### Authors’ and editorial staff’s experience with using the risk of bias tool

Table 3 presents the main results from the survey of review authors who had used the RoB tool, while Table 4 presents a summary of Cochrane Review Group staff responses to related questions, answered from an editorial perspective. Authors of all levels of experience with the RoB tool were represented in the survey (Table 3, Q1). The time taken to complete a RoB assessment for one trial varied widely among respondents, but the majority of respondents considered the time taken to be acceptable (Table 3, Q3 and Q4). We did not observe an association between number of reviews authored and reported speed of completing RoB assessments (χ^2^ = 18.9, *P* = 0.27). The majority of respondents (159, 84%) completed the recommended RoB table in RevMan, while 68 (36%) also included at least one RoB figure. The majority of respondents thought that the requirement to add quotes added transparency (128 authors and 49 editorial staff) and increased confidence in RoB assessments (104 authors and 30 editorial staff; see Additional file 1: Appendices 1 and 3).

**Table 3:** Extract of results from survey of Cochrane review authors who had used the risk of bias tool

**Survey questions**	**n (%)**
Number of reviews respondent used RoB tool in (Q1)	
One	80 (42)
Two or three	75 (40)
More than three	33 (18)
Used RoB tool to update an existing review (Q2)	102 (54)
Time taken to complete RoB assessment for one study (Q3)	
Up to 10 minutes	23 (12)
10 to 20 minutes	81 (43)
20 minutes to 1 hour	69 (37)
More than 1 hour	14 (8)
Time taken is acceptable (Q4)	156 (83)
Used pilot testing (Q5)	62 (33)
Modified the RoB tool when used for randomized trials (Q7)	56 (31)
Used the RoB tool for non-randomized studies (Q6)	39 (21)
Modified the RoB tool when used for non-randomized studies (Q6a)	31
RoB assessments incorporated in conclusion/analysis: (Q9)^a^	
Sensitivity analysis by RoB judgement	76 (40)
Included a narrative summary	104 (55)
Not at all	26 (14)
Used direct quotes to support judgement (Q10)	
Always or nearly always	76 (41)
Often	59 (32)
Feel confident in their RoB assessments (Q12)	
Very confident	61 (32)
Somewhat confident	111 (59)
Tool is better than previous Cochrane practice (Q13)	165 (87)
Features respondents most liked (Q14)^a^	
Ability to provide information (for example quotes)	140 (74)
Standardized approach	153 (81)
Features respondents least liked (Q15)^a^	
Judgement options (Yes/No/Unclear) confusing	69 (36)
Time taken to complete	56 (29)
Encountered problems with assessing sequence generation (Q17)	82 (44)
Encountered problems with assessing allocation concealment (Q18)	90 (50)
Encountered problems with assessing blinding (Q19)	94 (52)
Encountered problems with assessing incomplete outcome data (Q20)	122 (67)
Encountered problems with assessing selective outcome reporting (Q21)	110 (60)
Encountered problems with assessing other bias (Q22)	107 (58)
Other bias domain is helpful (Q23)	108 (61)
Use standard ‘other sources of bias’ (Q24)	53 (29)

Based on 190 respondents, authors who have used the RoB tool. Only the most frequent responses shown in the table, and some response options have been grouped to fewer categories. Not all respondents answered each question. Question numbers refer to the survey question in Additional file 1: Appendix 1. For full details of questions and responses, see Additional file 1: Appendix 1. ^a^ Respondents were allowed to select multiple answers for this question. *RoB*, risk of bias.

**Table 4:** Extract of results from survey of Cochrane Review Group staff

**Survey questions**	**n (%)**
Respondent’s role in the CRG (Q1)	
Managing editor	19 (33)
Coordinating editor	11 (19)
Other editor	11 (19)
Trial search coordinator/information specialist	2 (3)
Other	15 (26)
CRG policy regarding RoB assessments for new reviews (Q2)	
All new reviews must include RoB assessment	45 (78)
Recommended, but not compulsory	9 (16)
No clear policy or not sure	4 (7)
CRG policy regarding RoB assessments for updated reviews (Q3)	
All updated reviews must include RoB assessment	28 (48)
Only for newly included studies (Q3a)	3
Both newly and previously included studies (Q3a)	10
Recommended, but not compulsory	22 (38)
Only for newly included studies (Q3a)	0
Both newly and previously included studies (Q3a)	14
No clear policy or not sure	8 (14)
CRG staff verify assessments completed by their authors (Q4)	31 (53)
CRG recommend authors use pilot testing (Q5)	20 (35)
CRG recommend a modified RoB tool for randomized studies (Q7)	13 (23)
CRG recommend authors use RoB tool for non-randomized studies (Q6)	16 (28)
CRG recommend a modified tool for non-randomized studies (Q6a)	11
CRG recommend authors incorporate RoB in conclusion by: (Q9)^a^	
Conducting sensitivity analysis by RoB judgement	33 (57)
Including a narrative summary within interpretation of results	24 (41)
No specific recommendation	15 (26)
CRG recommend use of quotes to support RoB judgements (Q10)	34 (57)
RoB tool is better than previous Cochrane practice (Q12)	55 (95)
Features respondents most liked (Q13)^a^	
Ability to provide information (for example quotes)	48 (83)
Standardized approach	46 (79)
Features respondents least liked (Q14)^a^	
Judgement options (Yes/No/Unclear) confusing	24 (41)
Time taken to complete	20 (34)
Authors encounter problems with assessing sequence generation (Q16)	17 (29)
Authors encounter problems with assessing allocation concealment (Q17)	29 (50)
Authors encounter problems with assessing blinding (Q18)	33 (59)
Authors encounter problems with assessing incomplete outcome data (Q19)	41 (72)
Authors encounter problems with assessing selective outcome reporting (Q20)	38 (67)
Authors encounter problems with assessing ‘other bias’ (Q21)	32 (56)
Other bias domain is helpful (Q22)	27 (47)
CRG recommend standard ‘other sources of bias’ (Q23)	10 (17)

Based on 58 respondents, Cochrane Review Group staff. Only the most frequent responses shown in the table, and some response options have been grouped to fewer categories. Not all respondents answered each question. Question numbers refer to the survey question in Additional file 1: Appendix 3. For full details of questions and responses, see Additional file 1: Appendix 3. ^a^Respondents were allowed to select multiple answers for this question. *CRG*, Cochrane Review Group; *RoB*, risk of bias.

Nearly a third of respondents (56, 31%) said they had used a modified version of the RoB tool to assess randomized trials (Tables 3 and 4, Q7). Modifications consisted of adding new domains, modifying criteria for ‘Yes/Unclear/No’ judgements, or removing some domains. These modifications were usually based on own expertise (37 respondents), or following guidelines from their Cochrane Review Group (21 respondents; see Additional file 1: Appendix 1). Thirty-nine (21%) respondents had used the RoB tool to assess non-randomized studies, and 16 editorial staff who responded (28%) stated their review group recommended this practice. When used for this purpose, the RoB tool was usually modified (Tables 3 and 4, Q6). Non-randomized study designs identified by respondents were quasi-randomized, cohort, case-control, cross-sectional, interrupted time-series and controlled before-and-after studies. Modifications were usually based on respondents’ expertise and literature, but with no consistent or standard approach. Two other instruments reported to be used for this purpose were the Newcastle-Ottawa scale [10,11] and the Cochrane Effective Practice and Organisation of Care (EPOC) Group’s quality assessment checklist (see Additional file 1: Appendices 1 and 3) [12].

The survey responses indicated that authors need clearer guidance on what to do with RoB assessments once completed: 26 (14%) respondents did not incorporate their RoB assessments into review conclusions at all, while the majority (104, 55%) opted to include a narrative summary (Table 3, Q9). In terms of review group policy, the most prevalent recommendation was that authors should include a sensitivity analysis (Table 4, Q9).

#### Issues specific to individual bias domains

Authors reported some difficulties in completing each bias domain, but the domains thought to be most difficult were ‘incomplete outcome data’ and ‘selective outcome reporting’ (Table 3, Q17 to Q22). Editorial staff identified similar issues (Table 4, Q16 to Q21). Nevertheless, 172 (91%) of respondents reported feeling ‘somewhat’ or ‘very confident’ in their RoB assessments (Table 3, Q12). We did not observe an association between the number of domains with which respondents reported problems and whether or not they had any RoB-specific training (T = 0.29, *P* = 0.77). Similarly, having received specific RoB training was not associated with the respondents’ level of confidence in their RoB assessments (T = 1.59, *P* = 0.11). We describe below more detailed responses for each domain (shown in Additional file 1: Appendix 1).

The most common problems with assessing sequence generation were: confusing sequence generation with allocation concealment (50% of those reporting a problem with this domain); and difficulty in assessing whether a particular reported method was associated with bias (52% of those reporting a problem). Respondents also reported that the method of sequence generation was commonly not described in trial reports and accordingly wanted guidance on how to make judgements based on their overall impression of trial conduct. Similarly, if allocation concealment is well described and adequate, respondents wanted guidance on whether this can be used as a basis for a judgement of low risk of bias for sequence generation. Most respondents reported that they simply select ‘unclear’ whenever study reports do not describe sequence generation.

The most common problems with allocation concealment were: difficulty in assessing whether a particular reported method was associated with bias (61% of those reporting a problem with this domain); confusing allocation concealment with blinding (34% of those reporting a problem); and consistency between assessors (26%). Again, a commonly raised issue was insufficient information in the trial report, especially for older studies.

Respondents who reported problems with blinding experienced difficulty with making a judgement in studies where patients and/or caregivers cannot be blinded (68% of those reporting problems), while 64% reported difficulty in making a global assessment of blinding of patients, providers and outcome assessors.

The most common problems with the incomplete outcome data domain included: difficulties in making an assessment when the dropout rate is described but not acceptable (55% of those reporting a problem); establishing whether an intention-to-treat analysis had been conducted (57%); establishing what constitutes ‘complete’ outcome data (67%); making assessments of missing outcome data at different follow-up periods (52%); and confusing incomplete outcome data with selective outcome reporting (33%). Inconsistency in the meaning and understanding of the phrase ‘intention-to-treat analysis’ was also cited as a source of problems in some free-text answers.

The most common problem reported for selective outcome reporting was making an assessment without access to a study protocol (86% of those reporting a problem) and confusing selective outcome reporting with incomplete outcome data (41%). Inconsistency between assessors (20%) and lack of standard outcome measures in a given clinical area (22%) were also reported. One respondent raised concerns that this domain is not relevant to review results because either the missing information can be obtained from the study author, or the study cannot be included in the meta-analysis and should thus be excluded from the RoB table.

Many respondents (95, 89% of those reporting a problem with this domain) found it difficult to decide what should be considered under other sources of bias. Some suggested the domain is too vague and therefore open to misuse. The following are some of the items respondents had included under the ‘other bias’ domain in their reviews: compliance; baseline comparability; funding source and conflict of interest; adjustment for confounding factors; biases in cluster-randomized trials; carry-over effects in cross-over trials; co-interventions; early stopping of trials for benefit; multiple interim analyses; sample size calculations; publication bias; selection/recruitment bias; validity of outcome measures; surgical learning curve; and timing of outcome assessment. A decision on what should be included in the ‘other bias’ category had usually been made in consultation with co-authors (39 respondents).

Responses relating to training specific to the RoB tool are shown in Table 2 for all three groups of respondents, separately. Existing training materials and opportunities seem to be satisfactory in general, but respondents did favour provision of additional examples and web-based training.

### Recommendations and implementation

At the face-to-face meeting in March 2010, 23 participants considered the findings of the focus groups and the surveys and made consensus-based recommendations for improvements to the RoB tool, which are summarized in Table 5. Some of the short-term changes were implemented in a new edition of the *Cochrane Handbook*[8] and RevMan version 5.1 [13]. Specifically, wording of bias judgements was changed from ‘Yes/No/Unclear’ to ‘Low/High/Unclear’ risk of bias; category headings were introduced for selection, performance and detection, attrition, reporting, and other bias; authors are now encouraged to make separate judgements for blinding for 1) participants and personnel, and 2) outcome assessment; and guidance was clarified, particularly for incomplete outcomes, selective outcome reporting and ‘other sources of bias’.

**Table 5:** Summary of the panel recommendations and their implementation to date

**Recommendations**	**Implementation of recommendations**
Change the wording of bias judgements from ‘Yes/No/Unclear’ to ‘Low/High/Unclear risk of bias’	Implemented in RevMan version 5.1 and *Cochrane Handbook* version 5.1.0
Introduce category headings for selection, performance and detection, attrition, reporting, and other bias	Implemented in RevMan version 5.1 and *Cochrane Handbook* version 5.1.0
Split the assessment of blinding into: 1) participants and personnel; and 2) outcome assessment	Implemented partially in RevMan version 5.1 and *Cochrane Handbook* version 5.1.0. Full structural implementation scheduled for RevMan version 6
Clarify guidance, particularly for incomplete outcomes and selective outcome reporting, and ‘other sources of bias’	Guidance improved in *Cochrane Handbook* version 5.1.0. Further guidance development ongoing
Produce clearer and more explicit guidance on incorporation of RoB assessments into meta-analyses	Further guidance development ongoing
Weight RoB graphs by study size	Scheduled for RevMan version 6
Provide an algorithm for reaching a summary assessment of risk of bias per study/outcome	Working group established in 2012 to develop RoB tool 2.0 with signalling questions introduced into the tool to help guide assessors to make a domain-based judgement in a more structured way
Develop online guidance and training materials including an online frequently asked questions and a bank of worked examples of assessments	Working group tasked with the development of RoB tool 2.0
Assess how frequently Cochrane Review Groups include non-randomized studies in their reviews	Survey completed in 2012 as part of the development of the RoB tool for non-randomized studies
Develop a RoB tool for the assessment of non-randomized studies	The development of the RoB tool for non-randomized studies started in March 2012 and is expected to finish by the end of 2014

*RoB*, risk of bias.

Medium- and longer-term recommendations (implementation to coincide with the development of RevMan version 6 or later) include: separation of assessments of blinding into blinding of participants and personnel (under performance bias) and blinding of outcome assessment (under detection bias) will be enforced by structural changes in the software; weighting RoB graphs by study size; providing an algorithm for reaching a summary assessment of risk of bias per study/outcome; and developing a RoB tool for assessment of non-randomized studies. Extensions to the written guidance will be incorporated into upcoming versions of the *Cochrane Handbook*, including: further clarification of guidance with regards to selective reporting and other sources of bias; clearer and more explicit guidance for incorporating RoB assessments into meta-analyses; an algorithm for formulating summary assessments across domains of bias; and a bank of worked examples. A dedicated steering group was formed in 2011, funded by the Cochrane Collaboration’s Methods Innovation Fund, to develop a RoB tool for the assessment of non-randomized studies. This work is expected to be completed by the end of 2014. Another working group, formed in 2012, was tasked with introducing signalling questions within each bias domain and an overall RoB judgement for each outcome in the RoB tool for randomized trials in order to provide a more structured framework for reaching domain-level and outcome-level judgements. The same structure of signalling questions and bias domains is being implemented in RoB tools for randomized and non-randomized studies, with the aim of applying the same standards of assessments for all study types.

## Discussion

Our multi-staged evaluation of the RoB tool found wide acceptance of the need for the tool, with consensus that it represents an improvement over methods previously recommended for use in systematic reviews. The interpretation of these findings should however be cautious, due to a low response rate of the survey. The time required to complete assessments of risk of bias was greater than had been required by previous approaches, but was nonetheless considered acceptable. A high proportion of respondents reported problems with each of the individual RoB domains. The domains reported to be the most difficult to assess were risk of bias due to incomplete outcome data and selective reporting of outcomes. There was wide variation in how review authors had approached the ‘other bias’ domain, with a lack of clarity over what additional items should be considered here. Some of the items that authors have included (such as sample size calculations and funding source) are explicitly discouraged in the *Cochrane Handbook* guidance. While there is evidence that some factors are empirically associated with effect estimates, such as single versus multicentre design, early stopping of trials and funding source [14-16], the extent to which these should be considered alongside the main bias domains is still a topic of debate.

The evaluation highlighted a need for more and better training and guidance materials, such as algorithms or similar structured guidance for reaching domain-level judgements, as well as guidance on how to incorporate RoB assessments into meta-analyses and review conclusions. Recommendations for changes or further developments were made based on identified needs and many have already been incorporated into the new edition of the *Cochrane Handbook*, while other developments are underway. As suggested by evaluation participants, an online bank of worked examples for RoB assessments will be incorporated into future versions of the *Cochrane Handbook* or made available online.

This was the first study to evaluate the implementation of the new Cochrane tool for assessment of trials included in reviews. We used qualitative methods (focus groups) to help design the questionnaire, which we piloted to improve face validity. The focus groups were facilitated by the authors (DM, JACS, JS or LW), two of whom are bias experts and contributed to the development of the original RoB tool (DM and JACS). It is possible that, under such circumstances, the participants could have been reluctant to admit lack of understanding or confusion with the tool. However, the main purpose of the focus groups was to inform the development of the survey questionnaire and not to draw any firm conclusions. Some of the focus group participants were later involved in the piloting of the questionnaire. Although the proportion of respondents to the survey was small (4.4% of the 7,368 mailing list subscribers), it is possible that the effective response rate was somewhat higher due to a combination of overlap among the four mailing lists and the presence of inactive Cochrane review authors on the authors’ list. However, given the low response rate, it is possible that authors and Cochrane Review Group staff who read the email and chose to respond differ from those who did not read the email or chose not (or forgot) to respond. Due to time limitations, our survey was live for only 3 weeks, which also could have reduced the response rate. Nevertheless, the main purpose of this evaluation was to identify potential problems with the RoB tool that can be rectified, and we suspect that users who encountered problems are more likely to have responded. This speculation is based on the high proportion of respondents who reported having problems with some aspects of the RoB tool, especially with individual RoB domains. However, it is equally possible that those users of the RoB tool who experienced the most problems with RoB felt disillusioned and chose not to participate. One further limitation to consider is that the survey measured confidence and self-reported difficulty; it is possible that the number of people incorrectly applying these concepts may be higher as authors may be unaware of their misunderstandings. We also wanted to gauge general perceptions of users of the RoB tool, and to find out if their training needs were being met. Another potential limitation is the small number of non-users of the RoB tool represented in the evaluation. It is impossible to determine whether the number of non-user respondents was small because few authors made a decision not to use the tool or because such authors chose not to respond to the survey.

We are not aware of a similar survey of Cochrane review authors or evaluation of the RoB tool. Several studies used other methods to investigate the use of the RoB tool in practice and evaluate its reliability. Hartling *et al*. found that, although the tool takes longer to complete than other approaches, trials assessed to be at high risk of bias produced more exaggerated effect estimates compared to low risk trial reports [17]. This is consistent with other empirical studies [2,18]. The same authors assessed the reliability of the tool and found, consistent with the results reported here, that incomplete outcome data and selective reporting are the most difficult domains to assess [17]. It is important that guidance and training materials continue to be developed for all aspects of the tool, but particularly these two items. One of the findings from our evaluation that was of particular concern is that 44% or more of respondents had difficulty with assessing each of the individual RoB domains. This is consistent with the results of the reliability testing reported by Hartling *et al*. [17]. Inter-rater reliability is a substantial problem facing the RoB tool, in common with many of the other tools used for similar purposes in systematic reviews. Nevertheless, a further study has found the reliability of the RoB tool to be better when review-specific guidance was used, with reported agreement on bias domains ranging from fair to almost perfect [19]. Liu *et al*. carried out a review of systematic reviews of acupuncture in Chinese journals in the period from 2009 to 2011 in order to assess the prevalence of use of the Cochrane RoB tool in this field of research. They found that only 6% of reviews reported information on all six RoB domains [20].

Our evaluation led to recommendations for improvements to the tool [9]. There was consensus that assessment of blinding should be separated into blinding of participants and health professionals (performance bias) and blinding of outcome assessors (detection bias), and that classification of bias domains into categories of bias (selection bias, performance bias, detection bias, attrition bias, reporting bias and other bias) would be helpful. Some of the recommended changes have been implemented in RevMan version 5.1 [13] and in a revised version of the *Cochrane Handbook*, released in March 2011 [8]. There was agreement that improved training materials and availability of worked examples would increase the quality and reliability and reduce misuse of items assessed in RoB assessments.

The current RoB tool addresses main sources of bias in randomized trials of a standard parallel-group design. The evaluation helped to identify a need for timely development of extensions of the RoB tool to cover other randomized trial designs, and non-randomized studies. The next generation of the tool will meet the need for more structured guidelines for reaching domain-based RoB judgements (for example algorithms), since it will introduce a signalling question-based approach as used in the QUADAS 2 tool for assessing diagnostic accuracy studies [21]. Signalling questions are additional, specific questions within each bias domain aimed at helping the assessor reach the domain-level judgement more easily and in a more structured way.

More empirical evidence is needed to further inform considerations of what methodological aspects are most important in assessing risk of bias. There is a particular need for assessment of the influence of participant attrition on effect estimates, and on separate contributions to bias from blinding of patients and caregivers versus blinding of outcome assessors. Further, clearer guidance, ideally based on empirical evidence, is needed on how to deal with studies at high risk of bias in meta-analyses, other syntheses of evidence across studies and drawing conclusions.

## Conclusion

Our evaluation of the Cochrane RoB tool suggests that it is a step in the right direction, but that revisions of the tool and associated guidance, and improved provision of training, are required. Extensions of the tool for non-parallel group randomized trials and non-randomized studies were identified as a priority and such developments have been initiated as a consequence of this evaluation.

## Abbreviations

CRG: Cochrane Review Group; EPOC: Effective Practice and Organisation of Care; RoB: Risk of bias.

## Competing interests

JACS, DGA, DM and JPTH contributed to the development of the original RoB tool and continue to be involved in the tool development as well as the development of the extensions of the RoB tool for non-randomized studies and trials with non-standard designs. JS and LT are involved in the development of the extensions of the RoB tool for non-randomized studies and trials with non-standard designs. LW declares no competing interests.

## Authors’ contributions

JS participated in study design, conducted focus groups, administered the survey, analyzed the survey results, organized the face-to-face meeting, interpreted results and drafted the manuscript. LW participated in study design, conceived, conducted and analyzed focus groups, administered the survey, interpreted results, and helped draft the manuscript. JACS conceived the study, participated in its design, conducted focus groups, interpreted results and critically reviewed the manuscript. LT administered the survey, interpreted results and critically reviewed the manuscript. DGA interpreted results and critically reviewed the manuscript. DM conducted focus groups and critically reviewed the manuscript. JPTH analyzed the data, interpreted results and helped draft the manuscript. All authors read and approved the final manuscript.

## Supplementary Material

Additional file 1 **Appendices 1, 2, 3 and 4.** Evaluation of the Cochrane Collaboration’s tool for assessing the risk of bias in randomized trials: focus groups, online survey and proposed recommendations. Detailed survey and focus group results. Click here for file
