# Supplementary material for: Evaluation of the Cochrane Collaboration’s tool for assessing the risk of bias in randomized trials: focus groups, online survey, proposed recommendations and their implementation
Source: Syst Rev. 2014 Apr 15;3:37. doi: 10.1186/2046-4053-3-37 (PMC4022341; doi:10.1186/2046-4053-3-37)
Supplement: Additional file 1 — Appendices 1, 2, 3 and 4. Evaluation of the Cochrane Collaboration’s tool for assessing the risk of bias in randomized trials: focus groups, online survey and proposed recommendations. Detailed survey and focus group results. [file 2046-4053-3-37-S1.pdf]

Evaluation of the Cochrane Collaboration's tool for assessing the risk of bias in randomized trials: focus groups, online survey and proposed recommendations

Appendices

Table of contents

Appendix 1 – Risk of Bias Tool Evaluation: Survey of Authors: Users of ROB	2
Appendix 2 – Risk of Bias Tool Evaluation: Survey of Authors: Non-users	31
Appendix 3 – Risk of Bias Tool Evaluation: Survey of Editors & CRG staff	36
Appendix 4 – Risk of Bias Tool Evaluation: Focus Group Summary	58

Appendix 1 - Risk of Bias Tool Evaluation: Survey for Authors: Users of ROB

Detailed Results

Survey Overview

Number of respondents: 190
Launch date: 01 Feb 2010
Close date: 22 Feb 2010

The results from the survey are presented below question by question.

Section 1: Part A: Use of Risk of Bias Tool

**1.** In approximately how many reviews have you used the Risk of Bias tool?			
1 review:		42.3%	80
2-3 reviews:		39.7%	75
4-5 reviews:		8.5%	16
More than 5 reviews:		9.0%	17
I can't remember:		0.5%	1

**2.** Have you ever used the Risk of Bias tool to update an existing Cochrane review?			
No.:		46.0%	87
Yes. If YES,:		54.0%	102
**2.a.** For your update, did you implement risk of bias assessments for:			
Only new studies included in the update:		6.9%	7
Both new and original studies included in the update:		93.1%	95
I do not remember:		0.0%	0

**3.** Approximately how much time, on average, does it take you to complete a risk of bias assessment for each included study?			
Less than 10 minutes:		12.3%	23
Between 10 and 20 minutes:		43.3%	81
Between 20 minutes and 1 hour:		36.9%	69
Between 1 and 2 hours:		4.3%	8
More than 2 hours:		3.2%	6

**4.** The amount of time it takes to complete a risk of bias assessment is:			
Acceptable (i.e. the workload is balanced by the perceived benefit):		83.0%	156
Unacceptable (i.e. the workload does not justify the perceived benefit):		9.0%	17
I do not know / I am undecided:		8.0%	15

**5.** Have you ever incorporated pilot testing (for example, initial inter-rater testing, or testing decision rules) into your Risk of Bias assessments?			
No:		67.0%	126
Yes. If YES,:		33.0%	62
**5.a.** What did your pilot testing entail?			
Assessments by more than one reviewer:		n/a	58
Modifications to the approach suggested in the Cochrane Handbook or by your review group:		n/a	12
Explanation or elaboration of the approach suggested in the Cochrane Handbook or by your review group:		n/a	20
Other *(please specify)*:		n/a	2
Test-study + discussion with assessors			
to evaluate inter assessment differences			

**6.** Have you ever used the Cochrane Risk of Bias tool to assess the risk of bias for designs **other than randomized trials**?			
No. Please skip to question 7.:		79.1%	148
Yes. If Yes::		20.9%	39
**6.a.** Have you used a **modified** Cochrane Risk of Bias tool for this purpose?			
No:		20.5%	8
Yes:		79.5%	31
**6.a.i.** If yes, please specify how the Risk of Bias tool was modified			
Added new bias domains (e.g., a new domain about the similarity of treatment groups at baseline):		n/a	22
Deleted existing bias domains (e.g., sequence generation, blinding):		n/a	18
Modified criteria from the Cochrane Handbook for making judgments about bias domains:		n/a	13
Other *(please specify)*:		n/a	5
I have specified the above in protocols, but I can't remember if I ahve actually done this in reviews.			
Newcastle-Ottawa tool			
Samples characteristics in each group at each time interval Non-respondents characteristics within samples at each time interval			
sequence generation: stated as before and after allocation concealment: stated as before and after			
Used the EPOC RoB tools			

**6.b.** Please specify for what study design(s) you have used a **modified** Cochrane Risk of Bias tool			
Quasi-randomized studies:		n/a	18
Cohort studies:		n/a	19
Case-control studies:		n/a	8
Interrupted time-series:		n/a	3
Controlled before and after studies:		n/a	13
Other *(please specify)*:		n/a	3
Cluster randomised controlled studies			
cross sectional studies			
diagnostic studies			
**6.c.** Was your decision to use a **modified** Risk of Bias tool based on			
Guidance from your review group:		n/a	9
Your experience and understanding of bias-related issues for different study designs:		n/a	27
Literature around bias-related issues for different study designs:		n/a	19
Other *(please specify)*:		n/a	2
from another author of the review			
In studies in our CRG, blinding of participants and treatment providers is often not possible, so we are only interested in blinding of outcome assessors			

**7.** Have you ever used a **modified** Cochrane Risk of Bias tool to make assessments of bias of **randomized trials**?			
No. Please skip to question 8.:		69.1%	125
Yes. If yes::		30.9%	56
**7.a.** Please specify how the Cochrane Risk of Bias tool was modified			
Added new bias domains (e.g., a new domain about the similarity of treatment groups at baseline):		n/a	34
Deleted existing bias domains (e.g., sequence generation, blinding):		n/a	16
Modified criteria from the Cochrane Handbook for making judgments about bias domains:		n/a	22
Other *(please specify)*:		n/a	7
Added several categories of blinding to get more detail - participants, care givers, data collectors, was a sham surgical procedure done			
added subgroups of questions within a domain			

Differential expertise bias Validation of surrogate outcome
Free of other bias? -for cluster RCT only recruitment bias incorrect analysis
I was supposed to use the EPOC RoB tool for RCTs. I used the one described in the Handbook instead, because it was more explicit.
In the past we used another list with quality criteria
The Cochrane Drugs and Alcohol Group suggested to not assess the outcome reporting bias item because is very difficult to ascertain if protocol of studies are not searched

7.b. Was your decision to use a modified Cochrane Risk of Bias tool based on

Guidance from your review group:		n/a	21
Your experience and understanding of bias-related issues for randomized trials:		n/a	37
Literature around bias-related issues for different study designs:		n/a	14
Other *(please specify)*:		n/a	5

Instructions for the tem for selective reporting is a bit "fundamentalistic" for some areas
Need of other authors to describe different categories of blinding.
Some of the existing criteria (eg blinding) are just not practical for the educational nature of the RCTS in our review (smoking in pregnancy). Only 1/72 trials were "adequately blinded" using the Cochrane criteria. We also added biochemical validation of smoking status as research shows this has the largest effect on outcomes. The Cochrane statistician queried this and we changed the description from "low" ROB to "lowest"-to indicate that we are operating on a different scale. This decision was agreed by the authorship team, several of whom have lots of review experience.
The item deleted (blinding) was not applicable to the type of surgical trials included in the study.
to reflect issues related specifically to topic area

8. How have you reported Risk of Bias assessments in your Cochrane review(s)?

I completed the risk of bias tables:		n/a	159
I included one figure:		n/a	41
I included figure(s) and a table:		n/a	68
Other *(please specify)*:		n/a	9

described it in the text
I also summarised risk of bias for included studies in the text and discussed potential risk of other biases in each study in more detail.
Incorporated data within the descriptive summary
Narrative summary
Not yet completed
Review in progress - about to submit for edit process
Reviews done outside of the Cochrane
Some of this is at draft stage for the second review - for the first one the tables were sufficient as no meta-analysis was possible.
Two figures - one for bias in each study and second figure for overall bias across all trials.

**9.** How have you incorporated Risk of Bias assessments into a meta-analysis and/or conclusions of Cochrane review(s)?			
I conducted sensitivity analyses by risk of bias:		n/a	76
I restricted the primary analysis to studies at low risk of bias:		n/a	21
I included a summary within the interpretation of results:		n/a	104
I did not incorporate Risk of Bias assessments into a meta-analysis or conclusions of a review:		n/a	26
Other *(please specify)*:		n/a	12
I did not incorporate Risk of Bias assessments into a meta-analysis or conclusions of a review yet.			
Yet to include (in the process)			
I have not completed the review yet.			
Not there yet. Plan on using it for sensitivity analyses.			
Not yet decided.			
Still to agree with other authors what to include for the second review			
I discussed results with ROB in consideration			
I have stratified the analysis according to the Cochrane ROB score.			
I incorporated the RoB assessments in the conclusion			
The incomplete outcome data category determined which data were included. If not over 80% for a specific outcome it was not included.			
The study search returned only two trials. They both had several domains which were "unclear". We highlighted this in the discussion / results.			
unable to carry out meta-analysis			

**10.** How often do you include quotes (cut and paste) from included studies to support your risk of bias assessments in your Risk of Bias tables?			
Always:		13.4%	25
Almost always:		27.4%	51
Often:		31.7%	59
Almost never:		16.7%	31
Never:		10.8%	20

**11.** Do you feel the request for quotes to support risk of bias assessments			
Adds transparency to risk of bias assessments:		n/a	128
Increases confidence in risk of bias assessments:		n/a	104
Adds little value to the risk of bias assessment process:		n/a	19
Unnecessarily adds time to the risk of bias assessment process:		n/a	18
Other *(please specify)*:		n/a	10
Adds a lot of time			
Is very time consuming considering that it is not very useful afterwards.			
I find the quotations a real nuisance			
Adds transparency but at a great cost, that is, time. This is also reflected in my answer to question nr.3			
But it adds time, and for non-English articles it adds more translation time.			
However, ROB tool may assess how the reporting of the trial was done instead of how it was actually conducted due to poor reporting in the methods section of the publication.			
I can see the point of doing this, but sometimes it feels like hiding behind the coat-tails of the authors, OR implying well THEY said this, but um - can you believe them?			
increase understanding to the decision making			
Very dependent on the domain. If the text is completely missing, then quotes are not possible.			
Where the study has mentioned something which decreases the risk of bias (e.g. method of allocation concealment), quoting this allows you to more transparently put the trial in a low risk group. The difficulty is always in the reports which don't discuss methods adequately, as there's nothing to quote!			

**12.** How confident are you in your Risk of Bias assessments?			
Very confident:		32.4%	61
Somewhat confident:		59.0%	111
Not so confident:		8.0%	15
Not confident at all:		0.5%	1

Section 2: Part B: Your opinions about the Risk of Bias tool

**13.** Overall, compared to RevMan's past practice of recording only allocation concealment, the current recommended practice of assessing risk of bias is:		
Much better:	60.3%	114
Somewhat better:	27.0%	51
About the same:	2.6%	5
Somewhat worse:	1.1%	2
Much worse:	1.6%	3
I am unsure:	7.4%	14

**14.** Please specify aspects of the Risk of Bias tool that you **DO** like		
Ability to provide information (e.g., quotes from reports) to support a judgment:	n/a	140
Flexibility:	n/a	67
Good framework:	n/a	101
Standardized approach:	n/a	153
Table(s):	n/a	100
Figure(s):	n/a	96
Can be completed quickly:	n/a	49
There are no aspects of the Risk of Bias tool that I do like:	n/a	4
Other *(please specify)*:	n/a	10

Although it may not be the perfect tool for interpretation it would definitely lead to better reporting and the need to include quality of the trial in interpretation of study findings. Most Trial reporting tends towards over estimating the benefits of treatment and under reporting of adverse events.
based on empirical evidence
judgment instead of scores
makes me think more explicitly
outcome-based approach
separates out different categories of risk
Straight forward
The figures are great!
The info in the Handbook about reporting the findings from the ROB tool are skimpy and would benefit from being expanded
The standardized approach can also have drawbacks, such as in studies of physiological treatments for LBP(e.g. physical therapy, manipulative/manual therapy) because these treatments cannot be blinded, nor can the outcomes assessor be blinded. In our view if the patient is not blinded, neither is the outcomes assessor.

**15.** Please specify aspects of the Risk of Bias tool that you **DO NOT** like			
Time taken to complete assessments:		n/a	56
Table(s):		n/a	7
Figure(s):		n/a	12
Increased complexity:		n/a	40
Assessments are too subjective:		n/a	49
Request for quotes:		n/a	19
Difficult to modify:		n/a	26
The meaning of "Yes/Unclear/No" assessments is unclear:		n/a	69
There are no aspects of the Risk of Bias tool that I do not like:		n/a	30
Other *(please specify)*:		n/a	34

**Re: General comments and conceptual**
Assessments can still be subjective
Notwithstanding the use of quotes, it could still be "too" subjective. There's no getting around a certain amount of subjectivity and/or user error, I feel.
As with all such tools: they work to separate groups of studies with more and less bias but for a single study assessments might miss the point
Cannot allocate a score. Have been asked by reviewers of my systematic reviews for a score. I respond that there is no score
I think risk of bias complements the validity assessment well although it is time consuming - I think it is worthwhile as it gives a clearer summary of the weaknesses of studies included and therefore a balanced judgement can be made.
It lacks flexibility. For a clinical trial which has published several papers (different outcome indicators in separate publications), the RoB tool graph treats them as separate clinical trials and not belonging to a parent clinical trial. We feel that we do not get the correct proportion of bias at all for each of the domains. We brought this to the attention of an expert in the Cochrane group. *[The expert]* referred us to someone (forgot *[their]* name but I can look it up) and *[they]* admitted that it was a current limitation of the RoB tool.
Risk of bias changes and then also GRADE requirement added too much effort for my update, including having to go back to old studies. I also was requested after I did the scoring.
Several important types of bias are not pre-specified yet are mentioned in the handbook. If the risk of bias criterion has evidence to support its importance it should be included as a criteria in the RoB table.
Time invested is valuable to identify poor reporting or conduct of the trial or both and is very important (well spent) to interpretation of study findings.
it is very hard to teach people how to complete. often the most confusing part of reviewing for new authors
**Re: Bias assessment categories in ROB (Yes/No/Unclear) and unclear wording**
Expanding my response to the meaning of assessments, my concern is particularly with "unclear", which does not, I feel, distinguish between absence of information, and uncertainty about impact of reported information. This applies for example, to blinding in studies involving physical interventions.
I am uncertain re reporting bias - tendency to always select unclear. I am not sure that the tables and figures add much.
I feel I am using 'unclear' quite a lot - no more than in the past, possibly, and the fact I rarely say 'yes' probably reflects the type of reviews conducted.
If trials have different number and different timing of follow ups, there is not possible to select how many domains there is for each trial(e.g. for Incomplete outcome data). Íf data is missing for some domain of some trial the program assumes that the judgement is Unclear, although the domain may be irrelevant for that trial. However, Unclear and irrelevant are quite different things from the point of view of bias.
Some 'negative' questions such as 'baseline imbalance?' If baseline imbalance is low one would normally assume 'no', but this response would activate 'red'.
The yes/no answers are very confusing where there are double negatives

unclear how to deal with "unclear" categories at the analysis stage. Often when a methodological feature is not reported, one could guess (with a enough confidence) what was done
**Re: incorporating ROB assessments into meta-analysis / review conclusions**
It doesn't seem to have any influence whether bias is important in an included study. It will be included in the review however, and the fact that it is mentioned in the review that a RCT has bias is, in fact, irrelevant. it would be relevant if high risk of bias would exclude a study.
It is not easy to use a standardized approach to assess some items, e.g. selective outcome reporting, and to incorporate them in the analysis; it is not easy to decide when to use of overall vs domain-specific assessments in analysis
[Repeated comment]: unclear how to deal with "unclear" categories at the analysis stage. Often when a methodological feature is not reported, one could guess (with a enough confidence) what was done
**Re: RevMan and related issues**
Color coding make difficult reading in balck-white print
inability to automatically update data to RevMan tables.
should be an extra table (i.e. not attached to the 'characteristics of included studies table')
The Table in RevMan is a little bit unflexible to be modified and I find it annoying that a row disappears when you put unclear as judgement and do not put quotes.
Unable to alter the order of the domain in the table after one entry
**Re: Quotes**
It is very relevant that reviewers add quotes from reports. If they don't (for example only writing "none apparent source of bias" the quality of the tool is lost.
Sometimes requests for quotes are burdensome, sometimes they are helpful.
[Repeated comment]: The Table in RevMan is a little bit unflexible to be modified and I find it annoying that a row disappears when you put unclear as judgement and do not put quotes.
The tool itself is easy to use, but providing transparency from the original article requires sometimes an enormous amount of time, especially if it is an older study and text cannot be copied and pasted.
**Re: Domain-specific issues**
applicability to psychotherapy RCTs (issues of blinding, e.g.)
The question: Other sources of bias requires more guidance to the reader
**Re: Incomplete outcome reporting**
[Repeated comment]: If trials have different number and different timing of follow ups, there is not possible to select how many domains there is for each trial(e.g. for Incomplete outcome data). Íf data is missing for some domain of some trial the program assumes that the judgement is Unclear, although the domain may be irrelevant for that trial. However, Unclear and irrelevant are quite different things from the point of view of bias.
it does not provide a final summary of the quality of the study, therefore the decision on to which studies are of low quality (eg.for sensitivity analysis) is left to the author, and for the author is very difficult to decide
Its not clear how to deal with attrition. We assessed all trials which did not conduct intention to treat analysis as high ROB on that criteria. However, where possible, we adjusted for this and included all dropouts as continuing smokers in the CR meta-analysis. But there's no way of identifying on the "quick tables" which trials were adjusted in the review. We've just entered all the review data on to another software program (EPPI reviewer) and have added this dimension so we can assess the outcomes by true ROB due to lack of adjustment for ITT.
Presentation of incomplete outcome data could be clearer in the table eg introduce boxes to enter n values for each treatment group to show number of people randomised compared to number of people analysed for each outcome, This would seem more transparent to readers and would enable the review author to be clearer about the potential impact of incomplete data across separate outcomes.
**Re: Selective outcome reporting**
Sometimes older RCTs difficult to accurately assess as methods/protocol information is brief. All specified outcomes maybe reported but no clue as to what may have been omitted.
the assessment of selective outcome reporting and other sources of bias are very subjective.

**16.** Do you have any suggestions to make the Risk of Bias tool easier to implement in Cochrane reviews?
**Re: General and conceptual comments**
Could be made even more specific.
Risk of Bias need to be implemented in a different way for different topics (e.g. drgs and alcohol, EPOC,etc.) I suggest that Groups should be encouraged to provide Templates or detailed instruction on how to operationalize Risk of Bias tool according to specific characteristics of the clinical area under investigation
Having different tools for different study designs would be very helpful
needs to be adapted for the reliability of outcome measured eg death versus disease progression
Needs more flexibility
Make it easier to modify or adapt to specific review topics.
Perhaps simplify to the 4 main domains of bias: selection, performance, detection, attrition
Simplify it with fewer domains
Probably to eliminate some domain (such as publication bias) very difficult to assess
See above (15)- maybe an option to identify if you have adjusted the trial report in the CR analysis?
Does it really add anything to a review beyond a lot of work for the reviewer?
Remove *[ROB altogether]* - is too subjective, difficult to interpret for readers and comes down to author interpretation anyway.
**Re: Suggestions for rankings, scores, algorithms and scales etc.**
Although the risk of bias tool is a good way to assess RCT, most of them are finally rated as "unclear" even if there are substantial differences among them. I have been thinking about it and I propose to use non-conventional logic such as the "fuzzy logic" that could be very helpful to increase the power of discrimination of the tool. The only difference is to establish along with the current judgment a numerical scale for each domain from 0 to 100. For example for allocation concealment you could rate, even if "unclear", the % of confidence that the evaluator has that the allocation concealment was adequately performed. Later, this information along with the data from each domain, could be treated using fuzzy logic algorithms rather than the usual rates. This type of logic is similar to the way we usually think. An interesting study could be to compare the general "impression" of a reviewer reading a RCT to the final rate of the risk of bias tool (using and not the proposed fuzzy logic).....
Some sort of scoring framework. Eg low risk of Bias if there are no NO scores...moderate risk of there are 2-4 or more NO scores...high risk if there are 4 or more NO scores...
There is no ranking scale for ROB tools. For example, all trials are considered high-risk even if only one item is "unclear" or "NO", while the trials having only item "unclear" or "NO" are not equal to trials with two or more items "unclear" or "No". I suggest a ranking scale to categorize the high-risk trials as low-, medium- and high-risk.
some means of objective scoring which equates the present means of assessment
please provide a final classification score of the study quality
**Re: Bias assessment categories in ROB (Yes/No/Unclear) and unclear wording**
1. The wording in the tool is somewhat incongruent: The question are addressing quality but the tool addresses risk of bias 2. The presentations in the figure is not very nice for large reviews
Consider how to split "unclear" into "possibly yes" and "possibly no"
Elimination of 'negative' questions.
Including quick links to the meaning of Yes/Unclear/No under each item of the tool
Instead of Yes/Unclear/No just have low risk/unclear risk/high risk. It would be helpful to have a tool/calculator/further guidance to determine when incomplete data is a potentially significant source of bias and if so, how this could impact on the reported results. Perhaps if you had risk of bias graphs for each outcome in the review this would be easier to incorporate risk of bias assessments as you write the results for each outcome.
Replace Yes/No with Low risk/High risk

reword to adequate/unclear/inadequate
The yes and no are sometimes confusing.
**Re: Incorporating ROB assessments into meta-analysis / review conclusions**
Guide with examples for how to interpret findings when sensitivity analyses not possible. Selective and incomplete reporting of data in primary studies often restrict what can be done with respect to analyses/sensitivity analyses.
**Re: RevMan and related issues**
As I mentioned in item #15, the RoB tool should be able distinguish publications belonging to a parent clinical trial so that percentages appearing in the RoB graph will be true representations of the actual bias per domain. Unless this is corrected, we feel that percentages of bias become inflated or falsely elevated.
I fear that authors may not bother to look up the meaning of some of these bias-assessment terms. Could RevMan have a built-in glossary for certain terms one may encounter whilst actually doing things in RevMan? (like a link, from terms that could easily be misunderstood)
Instead of making about half a 'self-build' software we should rather have all bias mechanisms prearranged and the authors can deselect if the want to skip one or more.
1. Copy across option for multiple outcomes with same information. 2. Copy ability for studies with identical information. 3. Reject need for quotes
[Repeated comment]: Make it easier to modify or adapt to specific review topics.
monochrome options rather then traffic light colours for figure. proportion data to appear on figure
possibility to add components with title instead of lumping them in the other biases box
The table function in RevMan needs to be improved and becomme more flexible and user friendly. risk of bias is assessed on the level of outcomes but in RevMan, one can only produce one ROC for one study.
To attach grade software in the RevMan, to automatically update data.
would be helpful to have some pop-up notices within Revman to save constantly referring back to the Cochrane handbook. This would be helpful for reviewers.
**Re: Training, Guidance, Handbook and more examples**
I was really helped in using ROB tool when I attended a Review Completion Workshop and *[a tutor]* sat wit me and we did a ROB on the same paper together and discussed it. Although I had followed the advice in the Handbook, *[the tutor]* made it all much clearer to me.
No. I am too un-tutored in the whole process of reviewing in the Cochrane sense.
online training that can be taken as a refresher when doing the RoB
may be useful to include real time examples linked to actual studies as examples
More examples for each field (i.e. surgery, other fields)
More information the handbook - guidance with examples
This is a new topic and needs more training to access the data.
training and experience; examples and decision rules
**Re: Quotes**
Make inclusion of quotes optional.
*[Repeated section of the comment]: "…*3. Reject need for quotes…"
**Re: Domain specific issues**
There is overlap among the components of the RoB assessment. For example, concealment of allocation overlaps with masking. Correlation among RoB components makes it difficult to determine whether the overall "risk" is true.
Set up separate categories for blinding - participants, staff and those measuring outcomes
Sometimes the options are not clear cut yes, no, and uncertain. Example, if in blinding only the assessors were blinded it's difficult to choose any of the 3 options without compromise on the assessment of bias. There should

be a way of reporting the possible bias that may introduce to the review.
Use objective parameters to assess selective outcome reporting and other sources of bias.
Yes, include all types of RoB in RoB table eg Trial stopped early or multiple interim analyses or multiple endpoints or change of primary endpoint etc
To be thorough in doing a quality assessment, the existing tool is essential and once you get used to using it you can do it in less time. I would suggest adding in the Notes section any other biases (discrepancy in study data etc) that are identified by the authors/reviewers to be included.
Remove 'any other bias'

**17.** Have you encountered problems making assessments within the sequence generation domain:			
No.:		55.7%	103
Yes. If YES,:		44.3%	82
**17.a.** Was the problem related to			
Consistency between assessors:		n/a	19
Confusing sequence generation with allocation concealment:		n/a	41
Difficulty in assessing whether a particular method was associated with bias:		n/a	43
Adequately distinguishing between different non-random allocation processes:		n/a	16
Other *(please specify)*:		n/a	15
all of the above!			
Couldn't get sufficient information from trial authors.			
Data often not available esp in older studies			
For publications that do not explicitly mention this, we really have to write authors to extract this particular information. Sometimes it takes weeks before authors get back to us, if they get back at all.			
judgment depends on reporting quality			
Lack of information in papers, especially for older trials.			
Sequence generation is not often discussed adequately			
sometimes allocation concealment was specified but sequence generation was not...and how to interpret this?			
the main problem is studies merely reporting that they were randomised without describing how they randomised.			
What to do when only written randomised without any info about the sequence generation			
Some trials that have all the components of a well-designed trial (e.g., use of a placebo, use of identical packaging among interventions, masked outcome assessment, etc.) fail to describe whether or how randomization was used. Correspondence with the principal author was not possible, and correspondence with ancillary study personnel relied on their memory without written documentation. Is it justifiable to say that there was "no evidence of randomisation" in such a trial?			
whether "block randomization" can be assumed to be computer-generated; whether the use of the term "computer-generated" is sufficient to assume that it was not associated with bias			
Inexperienced reviewers often confuse sequence generation with allocation concealment. The main problem is that papers often don't state how they generated the random sequence			
Maybe it would be helpful if some examples were given, of adequate sequence generation processes were given, e.g. some block methods may apparently allow prediction, which I did not know			
unable to understand some of the methods described in some studies (ie statistical jargon)			

**18.** Have you encountered problems making assessments within the allocation concealment domain:			
No.:		50.5%	92
Yes. If YES,:		49.5%	90
**18.a.** Was the problem related to:			
Consistency between assessors:		n/a	23
Difficulty in assessing whether a particular method was associated with bias:		n/a	55
Confusing allocation concealment with blinding:		n/a	31
Other *(please specify)*:		n/a	15
all of the above!			
Data often not available esp in older studies			
the main problem is studies merely reporting that they were randomised without describing how they randomised.			
inadequate details in study			
judgment depends on reporting quality			
lack of information in papers (rather a general problem)			
Lack of information in papers, especially for older trials.			
Often not enough information reported to make a judgement on this			
often there is not enough detailed information			
papers rarely state whether they concealed allocation.			
This is often not discussed in trial reports			
The most frequent issue is inadequate information from the author to determine allocation concealment most commonly due to lack of precision around description of sequence.			
Poor documentation by original study authors of methods leading to many "unclear" assessments = difficult to draw conclusions about this domain.			
whether some simple phrases such as "centrally prepared drugs" or "randomization done centrally" are sufficient to assume that there was no bias			
Confusing sequence generation with allocation concealment			

**19.** Have you encountered problems making assessments within the blinding domain:			
No.:		48.1%	87
Yes. If YES,:		51.9%	94
**19.a.** Was this problem related to:			
Consistency between assessors:		n/a	14
Confusing blinding with allocation concealment:		n/a	13
Difficulty in making a global assessment of blinding of patients, providers and outcome assessors:		n/a	60
Difficulty distinguishing between double and triple blind:		n/a	17
Making an assessment for blinding when patients and/or providers cannot be blinded:		n/a	64

Other *(please specify)*:		n/a	22
all of the above!			
I have dealt with this problem by explaining my decision, which I thought was on of the benefits of the ROB tool, i.e. that it is transparent			
**Re: Lack of information in papers**			
Lack of information in papers, especially for older trials.			
and when the authors do not state whether it was blinded			
Issues of 'blinding' in caregivers of young children not always well described, and in cross age studies marking parent/carers or patients.			
Papers rarely state whether outcome assessors were blinded.			
Poor documentation by original study authors of methods leading to many "unclear" assessments = difficult to draw conclusions about this domain.			
**Re: Problems when blinding is not feasible or when unblinding is likely**			
Even when patients cannot be blinded, this may be a problem. I would therefore like to assess this as problematic.			
For surgical interventions blinding of care provider is often not feasible.			
As I work on surgical topics which cannot be assessed blindly, I sometimes am unsure whether to tick box "unclear" or "no". Most of the trials don't report on blinding, since it is so obvious that the trials were unblinded.			
Given the pharmacological effects of some drugs (e.g. benzodiazepines), sometimes it is difficult to determine whether blinding may have been broken, especially in placebo controlled studies.			
In some studies it is simply difficult (e.g. are trials of tricyclis against SSRIs really blind?)			
making judgments when blinding was not possible but outcomes shouldn't be prone to bias (e.g., mortality, information from administrative databases, blood tests, etc.)			
sometimes an issue between feasibility of blinding and possible to blind			
If some interventions are blinded and some are not. eg Oral sucrose, placebo and pink sugar solution. Can blind sucrose and placebo but not pink solution			
**Re: How blinding is addressed, by which categories separately etc.**			
At this moment there seems to be absolutely no consistency in assessment of blinding for the patient and outcomes assessor. I see in some reviews (and I apply this in my own reviews) that lack of blinding of the patient implies that the outcomes assessor is also not blinded (even though a blinded assessor might have been incorporated). However, in other reviews in a similar area, these 2 elements are considered separately.			
blinding should be assessed separately for patients, providers data collectors, outcome assessors and data analysts			
How to report when some are blinded and others are not			
I had difficulty with blinding when it was different for different outcomes and different for patients and providers in one study.			
In my case I split this question into 3: patients, care provider, and outcome assessor.			
Whether lack of blinding can affect a particular outcome - example all cause mortality, objective outcomes			
Relevance of assessor blinding for self-reported outcome measures			

**20.** Have you encountered problems making assessments within the incomplete outcome data domain:			
No.:		33.3%	61
Yes. If YES,:		66.7%	122
**20.a.** Was the problem related to:			
Consistency between assessors:		n/a	22
Overall complexity of guidance:		n/a	34
Difficulty in making an assessment when the drop out rate is described but not acceptable:		n/a	67
Difficulty establishing whether an intention-to-treat analysis has been completed:		n/a	70
Difficulty establishing what constitutes "complete" outcome data:		n/a	82
Difficulty making assessments of missing outcome data at different follow up periods:		n/a	64
Confusing incomplete outcome data with selective outcome reporting:		n/a	40
Other *(please specify)*:		n/a	14

all of the above!
Two questions in one: completeness and reporting
Difficulty in making an assessment when the drop outs are described but whether the reasons for drop-outs are acceptable
Papers often do not report the number enrolled and the number assessed.
Poor documentation by original study authors of methods leading to many "unclear" assessments = difficult to draw conclusions about this domain.
Guidance on 'incomplete outcome data addressed' overlooks the fact that there is empirical evidence that modified intention to treat analyses are potentially biased. Explaining exclusions after randomisation does not remove bias as suggested by the handbook and RoB table.
I think the evaluation of RoB from incomplete data is the most difficult. If proportion of missing data is unequal among study groups and no imputation has been used, then we should replace missings by high/low values to see if the results could be overturned. Should we check more for type I or type II errors? We checked only for type I (risk of saying an intervention is efficient when it is not). Guidance from my review group on this subject was absent. I was going too far for them. Also: It is difficult to evaluate if the imputation used is appropriate. I included a biostatistician to do so, but his opinion differed from the one presented in the missingdata.org website (referenced in the handbook). More guidance on imputation would be appreciated. I think imputation should be accepted up to a certain degree. This should be made more explicit.
In some cases it is simply difficult and subjective to decide whether there was bias...
this was the most difficult...with attrition, missing data, and ITT issues; this should be simplified!!
In some trials, outcome data may be complete but analysis is done on an as-treated basis. Intuitively, one would not assume "incomplete outcome data" to be present, but the analysis still is biased. Furthermore, it sometimes is not clear what post-hoc exclusions are justifiable (eg. retraction of informed consent, therapy not started, etc.)
inconsistent use of the term "intention-to-treat" across reports, hence it is difficult to determine what was done and whether a true ITT analysis was conducted
It is not easy to define the rules or criteria for the overall judgement for the domain of Incomplete outcome data considering all aspects for that.
Not all studies have all outcomes assessed; i.e. the number of included studies may vary by outcome. There should be a possibility to vote "not applicable"
see 15 above. Its good to report the trial results separately to assess "acceptability" of the program (ie if

lots of drop outs), but there's no way of specifying when you have adjusted the outcomes to include dropouts in the CR (as we have where we can in our *[anonymised]* review - ie where possible, all dropouts included as *[treatment failures]*.)

**21.** Have you encountered problems making assessments within the selective outcome reporting domain:			
No.:		40.5%	75
Yes. If YES,:		59.5%	110
**21.a.** Was the problem related to:			
Consistency between assessors:		n/a	22
Difficulty making an assessment without access to a study protocol:		n/a	95
Confusing selective outcome reporting with incomplete outcome data:		n/a	45
There being no standard means of measuring outcomes in your field:		n/a	24
Other *(please specify)*:		n/a	13
all of the above!			
Almost always unclear when no access to protocol. So not very useful in the tool...			
Unless the full protocol is available I can't see how this can be fully assessed			
This was especially problematic for clinical trials conducted prior to the time when registration in clinical trials registry became mandatory. Also, not many investigators will pass on their protocol just like that. This document is usually confidential and cannot be accessed readily by Cochrane investigators.			
What to do when get access to protocol in eg clinicaltrials but the dataform was started after the study was finished. Belive people report honestly or assess this as not having info from protocol.			
Authors would have to be very stupid to indicate in the manuscript that outcomes were collected but not reported. Seems pointless to do assess this. Going back to protocol is too time consuming.			
Difficult to judge no. In which cases should no be used? All included studies get the same argument: "There is no reason to suspect selective outcome reporting". It is difficult to give other arguments for judging yes.			
hard to judge what is missing, given expected variation in measures used and reported across trials			
Many trials do not report primary outcomes. Irrespective of whether the protocol was available or not, lack of reporting the primary outcomes should be considered selective outcome reporting.			
Studies with low methodological quality tended not to explain precisely the protocol. Sometimes we just have to assume some things and take conclusions based on incomplete information.			
To do this assessment properly I think an "outcome reporting matrix" needs to be done and this is time-consuming.			
We have used our own modification to assess reporting bias with no really clear feedback about whether this is acceptable from editors/reviewers. We look at whether a trial measures our review outcomes, whether a trial reports results but not data for outcomes, and whether a trial does not report results or data for an outcome they set out to measure			
What about selective outcome reporting if the outcome needed was delivered upon request by the author? What about obvious selective outcome reporting which does not concern the reviews outcome of interest. Generally, I feel this item is not really relevant to the review results - either I get the missing information from the author, or i can not include the study, and then it will not be in the RoB Table... Is it the current holy cow of the CC, after concealed allocation?			

**22.** Have you encountered problems making assessments with the other sources of bias domain:			
No.:		42.5%	79
Yes. If YES,:		57.5%	107
**22.a.** Was the problem related to:			
Consistency between assessors:		n/a	22
Difficulty determining what other types of bias to consider:		n/a	95
Other *(please specify)*:		n/a	19
all of the above!			
These are not so overwhelming in number that they could not be included specifically with specific definitions in RoB table.			
This is a vague, catch-all category. When all other components have had been rated has having "no" RoB, the tendency is to rate this component as "no" as well, especially for trials with brief methodology statements.			
This is highly subjective			
This is too vague- many authors are biased in what they put here			
yes, very difficult determining what, if any other types of bias exist			
You make enemies of your international colleagues when you score their publications unfavourably :-(			
Each trial will have different factors to consider and poor reporting magnifies the problem to detect other biases.			
I made a list of possible other biases from my readings (handbook mostly) and checked each item. I also read the discussion because authors often describe their own biases...:-)			
I think that "other bias" assessed should be specified in the method section. If not, this item is meaningless			
**Re: Specific issues addressed under this domain**			
Difficulty making judgments around source of funding and when that may create bias			
Difficulty in assessment of "funding" and "type of publication (i.e., abstract)"			
Dosages of drugs.			
For cluster randomised trials assessing risk of recruitment bias can be difficult			
How much compliance should there be to judge acceptable compliance? What does similar timing of outcome assessment means? I am always unsure here.			
In the assessment of "surgical learning curve bias" (aka differential expertise bias) standard methods to evaluate this type of bias are lacking.			
specific case of certain complex designs, like sequential, factorial and cross-over trials: how to assess bias			
When a baseline between-group difference is relevant?			
I include different types of studies so the problem of a missing n/a category remains			

**23.** Do you think the Other sources of bias domain is helpful to make Risk of Bias assessments?			
No:		38.6%	68
Yes:		61.4%	108
**23.a.** Please explain your response:			
**Those who responded NO:**			
Because of inconsistency between assessors. Better give examples.			
I like the basic idea of an item that opens for all the things not fitting in to the other items. But it is very			

difficult to decide what to assess.
I think it is more correct to include more domains (e.g. compliance) rather than using an "Other sources" domain. My experience is that the "other source" domain ends up as a domain that is used to push the overall risk of bias judgement in wanted direction.
I would prefer authors to state explicitly what other biases they are looking for.
It is a vague category. If we are going to assess bias, there should be distinct categories, and "other" should be avoided.
It is too subjective.
too general and not enough specific
Too subjective
No definition available
The category it too vague.
Lack of knowledge of the statistical basis of considering bias.
see #22 above
The heterogeneity from each reviewer impede summarize this bias
These are not so overwhelming in number that they could not be included specifically with specific definitions in RoB table. Lack of prespecified criteria leads to inconsistency in reviews.
**Those who responded YES**
Absolutely! Our most important source of bias (biochemical validation of smoking status) couldn't be included without an "other" section.....PLEASE don't take this out.
adds flexibility
Flexibility
Allows flexibility in the table to include context or topic-specific biases
Allows for further explanations within cross-over design trials
Allows sources of bias to be evaluated that are not applicable to other sections; such as limitations in sub group analysis with small participant numbers or use of non-validated outcome measure
Allows the review-specific other sources of bias to be assessed
As it adds an extra degree of sensitivity through flexibility.
bias depends on context so there will never be an 100% framework and 'other sources of bias' should remain
Bias due to publication ,language and grey literature etc could be judged here
But it should not be compulsory
But not very often.
can document declared sponsorship of trial (pharma influence)
Can give details of particular bias - e.g baseline imbalance associated with a particular study, or trial stopped early etc
default 'unclear', but sometimes evidence of additional bias
Different reviews have different sources of risk of bias.
Difficult as hard to know what this covers. feels like a 'catch all' but not sure what trying to catch. Assuming includes issue like detecting salami slicing?
For example if conflict of interest was not declared in the reports of the trial, then the results can be doubted. Also, if the researchers did not use a validated outcome measure, this may again compromise the results of the study.
give some flexibility if other methodological flaws

gives you a place to state other problems with a study that aren't covered in the provided categories
good to have an option for dealing with unforeseen problems
I found several other biases, even if it was not often. I think these should be acknowledged.
I often include information outlined by the author in terms of limitations (e.g. sample size bias)
I think other sources of bias domain is helpful; however, It is not clear how to assess and weight this domain.
I think this Other sources of bias domain makes your reader extra watchful and makes them consider conclusions asserted by individual studies more guarded because this domain takes into consideration what might not be readily apparent to readers. Take for example the bias exerted by early stopping of a trial. Before, even the most respected epidemiologist would not consider this a problem, especially in the presence of early stopping rules, but now, we learn that this in fact introduces bias.
If is specified in the method section which kind of other bias will be assessed
in our area sample size is a issue - this allowed us to factor this in.
Industry funding RCT need to be mentioned as a source of bias not included in the Risk of Bias assessments. Other commentaries en methodology, results or discussion of each RCT that in opinion of the assessors could induce a bias need to be registered in this sub-domain
It adds to make the assessment complete, e.g. to mention baseline imbalances.
It allows to describe and evaluate other kind of bias.
It allows to include other domain: academic bias, drug company bias, etc.
It is helpful because it adds information. Nevertheless it could be improved it this information was specified in specific domains, e.g. "baseline imbalance",...
It is helpful but needs more clear guidance.
It is useful to consider other sources of bias that are not already taken into account by the other domains- otherwise they'll get ignored as will their effect on study quality
It may help to give further insight into the overall level of bias with the trial especially if deciding on the level of bias in the main criteria is difficult.
It poses the question so that consideration must be given to other sources specific to the topics being reviewed.
it provides the opportunity to assess strange things or fatal flaws
It reminds us that other bias mechanisms may be operative.
It's helpful in capturing things like "source of funding". Need more guidance re: other sources of bias. This domain may look very different across reviews (inconsistent).
Learning curve effects are extremely important in surgery. Their effect often exceeds the effect of the intervention.
Many particularities can be found when assessing RCTs for methodological quality. Having the possibility to address these issues is a necessity.
Not all risks of bias are addressed in the tool.
Only if you specify what they are upfront, otherwise everything can be a potential bias in the eye of a reviewer.
Opportunity to address other sources of bias which are individual to a particular review/study other than the ones in the tool.
Particularly relevant for non-RCTs
Place to describe aspects of a study that might introduce bias but which are not easily fitted within other parts of the ROB table.
Please read my publication: *[Anonymised].* Other sources of bias can significantly alter treatment effect.
Potentially yes but not always easy to complete

relevant to assess other potential sources of bias
Some other sources of bias, like funding are very important and must be taken into account!!
Source of funding by pharmaceutical companies may bias the outcomes
The assessment of other sources adds information regarding possible problems that may not necessarily be associated with the standard criteria. Discrepancies between groups at baseline and funding influence are good examples.
the fact that there are many other types of bias such as funding bias, publication bias etc that might influence the overall result is very important
There are other sources of bias that do need to be considered.
There are some things we usually look for as standard in the 'other' section but it is unclear how important these aspects are. We tend not to use them in terms of integrating them into the review as much as the previous items.
This is one area that we find different reviewers can be inconsistent however it is useful to have the option for recording other problems with the trial
This question could have accommodated an "unclear" option. The important sources of bias seem to be well covered by the other standard options, but it is important to be able to report special issues.
Though subjective, this provides opportunity for the review author to state whatever other bias s/he may have noted in the paper that may impact on the outcomes
To have a place to write down some other feelings of bias
Useful catch-all category when dealing with CBA and ITS designs (for EPOC reviews)
Useful if more guidance provided. Other aspects can go here (e.g. Trials stopped early for benefit)
very useful to assess risks of bias that are inherent to a certain topic (e.g., attention bias in psychotherapy trials)
Yes although it depends upon how much 'weight' (subjective) you decide to give these other sources of bias. It is generally only one reviewer who comes up with the 'other' sources as the other co-reviewers tend to have clinical rather than methodological expertise therefore depends very much on the skills & experience of the review team.
Yes but tools for other designs would be helpful
Yes, but the success relies on an explanation in the text
Yes. Since publication bias, country of origin bias, language bias, cultural bias in interpretation of pain outcomes, conflict of interest bias all need to be accounted for.
You can describe your concerns about other sources of bias that have not been identified above.

**24.** Are there standard "Other sources of bias" that you tend to include in your Cochrane reviews?			
No.:		70.9%	129
Yes. If yes::		29.1%	53
**24.a.** Please specify what other sources of bias:			
"Compliance", "Paralell interventions avoided or identical between groups"			
- baseline comparability (especially in case of unadjusted analyses) - commercial funding			
- Early stopping of a trial based on conventional P less than 0.05. - Entry imbalance even if adequate methods have been used for randomisation. - Vested interest bias - academic or industry.			
1. Declaration of conflict of interest along with the funding adent. 2. Publication of the protocol of the trial to test compliance. 3. Validity of the outcome measures.			
academic bias, drug company bias.			
adjustment for confounding factors in open randomized studies methods of statistical analysis			

Baseline comparability Same therapists used across both conditions
Baseline differences, co-interventions, compliance and timing of outcome assessments. Where these all similar in both groups?
baseline imbalance
Baseline imbalance bias; early stopping bias; academic bias; source of funding bias. I am planning to include "Differential expertise bias" in my future reviews. I am also thinking about how to incorporate bias in the review due to choosing "surrogate outcomes" as primary outcomes
baseline imbalance, trials stopped early
baseline, co-intervention, timing of assessment
Bias for cluster studies: loss of clusters, incorrect analysis, recruitment bias
Co-interventions Baseline characteristics Funding
comparability of groups at baseline, contamination between treatment groups
Design-specific risks of bias, early stopping, inappropriate influence of funders
Early Stopping of a trial
early stopping of trial extreme baseline imbalance
factorial trials and the issue of synergistic effects
Free from obvious carry over effects? for crossover trials
Funding
Funding
Funding
funding aspects
funding bias, belief bias
Funding bias, publication bias, language bias,
funding.
in non randomised studies and the methods is not described properly or the design allows for other sources.
inappropriate influence of study sponsor; stopping early for benefit; baseline imbalances; inappropriate methods (usually statistical) for cross-over or cluster trials; inappropriate use of cross-over designs (e.g., condition changes over time)
industry bias, publication bias (hard to determine)
Industry funding studies and declaration of conflict of interest by the researchers. An incorrect statistical analysis for the data.
info on sample size calculations, premature stopping of trials, registration in trial databases
Inter and intra examiner reliability in assessing the outcome.
items specific for observational studies, it they ae included in the review
Multiple interim analyses. Trial stopped early. Multiple primary outcomes. Change in primary outcome.
publication bias trials stopped early
Referral, publication
Sample size - adequate in relation to power calculations
Sample size bias, information outlined by the author as potential limitations that could lead to bias,
Selection/recruitment bias
Small groups of participants with risk of being underpowered
Sometimes whether the clinical diagnosis was made using good methods.

Source of funding
stopping early for benefit
study stopped early, industry funding?
Surgical learning curve bias (= differential expertise bias)
Time between when the last patient was recruited and when the study was reported. Differences in numbers between data in abstracts from scientific meetings and the final publication.
Timing of outcome assessment
use of co-interventions compliance blinding of practitioners / patients / outcomes
usually report on funding of trials
vested interest bias
Whether funding source is reported and where funding comes from

**24.b.** How do you determine which other types of bias to include in your reviews?			
They are mandatory for all Cochrane reviews within my Cochrane review group:		n/a	6
They have been recommended by my Cochrane review group:		n/a	17
I determine them in consultation with other review authors for each individual review:		n/a	39
Other *(please specify)*:		n/a	7
based on literature on research methodology			
I just decided on my own (a sin I know)			
Literature			
Only for assessment of non randomised studies			
Own expertise.			
Own judgement, discussion with the group.			
read the handbook and guess what might be important.			

Section 3: Part C: Training related to the Risk of Bias tool

**25.** What type of training relating to the Risk of Bias tool have you participated in?		
I have attended at least one workshop at a symposium or colloquium:	n/a	74
I have attended at least one workshop independent of Cochrane symposia or colloquia:	n/a	32
I have attended Cochrane's standard author training:	n/a	44
I have read relevant training materials on my own time:	n/a	124
I have not received any specific training:	n/a	29

**26.** Have you read the guidance in the Cochrane Handbook related to the Risk of Bias tool?		
No. Please skip to question 27.:	5.8%	11
Yes. If yes::	94.2%	178
**26.a.** Please specify if you have:		
Read Chapter 8 Assessing Risk of Bias in Included Studies:	n/a	144
Read the Cochrane Handbook (Part 2) from start to finish:	n/a	30
Used the Cochrane Handbook to look up specific issues related to risk of bias:	n/a	147
Other *(please specify)*:	n/a	4
Discussed with fellow Cochrane authors who are experts		
read a selection of chapters in the handbook		
risk of bias, as other things, are not clearly explained, and Cochrane groups are usually very busy and not willing to put in time and explain. the usual answer is "it's in the handbook". I don't think that this indicates that enthusiasm and hard work are not very much appreciated.		
Used the publication: 2009 Updated Method Guidelines for Systematic Reviews in the Cochrane Back Review Group.		
**26.b.** Is the level of detail in the Cochrane Handbook related to the Risk of Bias tool:		
Not detailed enough:	21.9%	39
An appropriate level of detail:	74.7%	133
Too detailed:	3.4%	6
**26.c.** Do you feel the provision of further examples of risk of bias assessments would be beneficial?		
No:	20.2%	36
Yes. If yes::	79.8%	142
**26.c.i.** Would you recommend:		
General examples:	n/a	74
Examples specific to each Cochrane review group:	n/a	73
Examples specific to types of interventions (e.g. drugs, behavioral interventions, complex interventions):	n/a	103

Other *(please specify)*:		n/a	8
can all opens trails be judged as inadequate allocation concealment?			
I guess more examples especially with regard to complex interventions and examples from the EPOC review group would be helpful..			
Maybe more specific instruction about what to do with non-randomized studies (I know that there is some already)			
Other study design modifications			
specific examples regarding how to incorporate RoB assessments into analysis; more examples regarding selective outcome reporting, incomplete outcome data and other risk of bias			
Why is the current example not real?			
Would be helpful to have some 'health services research' examples for the type of reviews I do			
You could put in real life examples that have thought to have been done particularly well, perhaps linked to the relevant review and the pubmed id so we could look at the source report.			

**27.** Have you received any guidance from a Cochrane review group related to the Risk of Bias tool?			
No. Please skip to question 28.:		57.7%	109
Yes. If yes::		42.3%	80
**27.a.** Please describe that guidance:			
I was advised to read Chapter 8 Assessing Risk of Bias in Included Studies in the Cochrane Handbook:		n/a	43
I was given specific written guidance developed by my review group:		n/a	31
I was given specific verbal guidance by my review group:		n/a	33
I was advised to enroll in a workshop related to the Risk of Bias tool:		n/a	7
Other *(please specify)*:		n/a	5
I am the quality advisor of the *[Anonymised]* Group and I wrote the Template of my group which was further distributed to all reviewers			
I had a session with the CRG managing ed, making the RoB assessment together			
I received some important feedback from the Oral Health Group during the refereeing process of my 1st review.			
The *[Anonymised]* review group co-ordinator inserted the appropriate domains in the risk of bias table and the editors inserted the appropriate methodological text as our review was an update.			
When I have doubts in the risk of bias, I write to the group and the group co-ordinating editor provides with the assessment with the reasons for the assessment			
**27.b.** Do you feel that the guidance provided by your review group was:			
Excellent:		20.0%	16
Very good:		33.8%	27
Good:		30.0%	24
Fair:		11.2%	9
Poor:		5.0%	4

**28.** Do you feel the availability of training materials for personal use (e.g., Handbook and other written guidance) related to the Risk of Bias tool is:			
Insufficient:		19.5%	36
Sufficient:		75.1%	139
More than sufficient in relation to other required training materials for other topics:		5.4%	10

**29.** Do you feel the avaliability of training events (e.g. workshops) for the Risk of Bias tool is:			
Insufficient:		26.7%	46
Sufficient:		68.0%	117
More than sufficient in relation to other required training events for other topics:		5.2%	9

**30.** What format of training for the Risk of Bias tool would you be **MOST** likely to access?			
Training that is incorporated into standard author training:		17.1%	32
Online training, including webinars:		54.5%	102
In-person workshop: ½ day:		12.8%	24
In-person workshop: Full day:		10.7%	20
In-person workshop: Two days:		3.7%	7
Other *(please specify)*:		1.1%	2
Online or half day but prefer the Handbook anyway			
Training incorporated in colloquia			

**31.** What level of training would you be **MOST** likely to access:			
Beginning:		11.6%	22
Intermediate:		41.3%	78
Advanced:		47.1%	89

**32.** If you have any other comments related to the Risk of Bias tool that you would like to make, please record them here.
Although the handbook is excellent, i do feel that i would benefit from attending either a half or full day course to update my skills. There only seems to be courses for new reviewers rather than experienced reviewers who need updating or a short refresher course to be updated on the latest changes to methodology. Despite having been involved with Cochrane reviews for about 6-7 years now, i have not had the time/funding to attend the Cochrane Colloquia which is reportedly an excellent conference & ideal opportunity to be updated on the latest methodology. A short refresher or update workshop for experienced reviewers/editors would be great.
As empirical evidence for impact of specific bias becomes available, specific type of bias needs to be defined and specified in RoB table and then incorporated into training. Hierarchical methods for incorporating RoB into review should be specified by collaboration - eg report as primary outcome for only those trials meeting prespecified quality criteria.
As with all other aspects of Cochrane reviews, the methodology is now becoming so complex and time-consuming that it is no longer possible to leave authorship of reviews to enthusiastic amateurs. Reviewers need to have substantial time ring-fenced for performing the review. I am providing support and training to clinical reviewers for one CRG and, despite the free help that they receive, they make very, very slow progress with reviews and most of the work that they do needs to be done again because it is so weak. I feel that it is just not possible to do a Cochrane review round the edges of a demanding day job. However, in the early days of Cochrane, many people who are now consultants did just that, but the standard of the reviews they produced would now no longer be considered acceptable. Hence I think there are misconceptions among clinical reviewers about what is involved and the standards required. My experience with clinical reviewers makes me question the standard of education in evidence-based medicine provided by medical schools and the standard in specialist clinical journals, many of which rely on a small, closed network of clinicians for authorship and refereeing and do not have outside statistical or methodological help to raise standards.
At the moment the nuisance is greater than the perceived usefulness. Generally I am somewhat wary to disguise subjective decisions as objective / standardized.
For those authors conducting research with or reviews with the ROB tool currently, how will the changes to the tool (if any) impact the relevancy of those findings (e.g. Hartling, 2009)
Good work everyone!
I consider that the risk of bias tool should be able to assess how close the effect estimate obtained from the systematic review is to the true effect. The risk of bias tool is not complete at present. It misses very important issues such as differential expertise bias and the use of surrogate outcomes in reviews.
I don't have a feel yet for how obsessional it is desirable to be in trying to assess the risk of bias. As with all new methods the pioneers are all too liable to exaggerate its importance and to go over the top.
I don’t find the question for evaluating selective reporting useful. In most cases there is no published protocol, although admittedly this will become less of a problem in the future. However, I invariably score this as unclear and wonder if this isn't a greater problem in other review groups than my own.
I found the tool to be cumbersome to use initially but this improved greatly with experience. Examples would be very much appreciated.
I think it is a good tool and can be modified further to make sure trial reporting is done in a standard way to help future authors find information easily in publications. The Journal editors in their information for authors section could include quality assessment of each trial as a minimum requirement in describing methodology of each published trial.
I think it is a good tool that needs some modifications; this questionnaire is relevant and welcome.
I think it is excellent and has much improved the quality of our reviews. It has made it much easier to incorporate assessments of bias into the conclusions of the review.
I think it is great.
I would like to read in the Handbook and/or access online more practical examples of assessment of Risk of Bias by type of intervention, and not only in studies of drugs. I think the teory in the Handbook is OK but more examples would help assessors to make a more homogeneous opinion. Underlining options in "Other source of bias" as "Industry funding" "Conflict of interest declared by researchers" "Correct statistical

analysis" etc. would orientate assessors in their assessment.
If you are the author co-author of a large number of Cochrane reviews, updating these reviews and completing the RoB tables takes an extra-ordinary amount of time. Fortunately I have kept the original papers included in the reviews so I can go back, without having to retrieve the papers again.
Love it!
No, interesting development, but an enormous amount of extra work you demand from authors!
seems to be conflicting in different parts of chapter 8; one area describes it one way, then another area describes bias differently
Sensitivity analyses by level of risk of bias, like previous quality assessment tools, may be difficult when there is a limited amount of data available. I suspect this is common in many reviews (as they often conclude more evidence is necessary).
Some of these answers are irrelevant because I do not intend to undertake another review.
The help from the vascular review group was excellent.
The main problem is updating existing reviews!!!! When you have 9 previous reviews some with 40-60 trials this is an impossibility in the time one have available.
The painful process of completing it has made me seriously doubt I will undertake another Cochrane review.
The problem with the workshops comes when nobody pays for your training. In other words, I couldn't have access to workshops because I had no funding (I am not a MD).
The RoB tool can be developed similar to the Quality Assessment Tool for Quantitative Studies - EPHPP(Thomas et al 2009) to incorporate global rating of each study as strong, moderate, and weak.
The RoB tool is mis-named. It is not an actual "risk assessment" and, apart from leading to a sensitivity analysis (at least for the 'allocation method' component), does not lead to a modification of the overall 'relative effects' measurement of the combined trials. Furthermore, this tool further separates the systematic review author apart from the trialists, putting the meta-analyst into the role of both judge and jury over patients and physicians who have yielded the original data.
The tool is an improvement, but together with GRADE introduced too strictly. Be aware not to lose goodwill with your reviewers. Especially my *[Anonymised]* group is being strict.
This should not be compulsory in a Cochrane Review
With reviews that need updating and also IPD reviews that have been pre-published elsewhere completing the risk of bias tool retrospectively for each has been onerous. There seems to be inconsistency across CRGs in this regard and possibly in their understanding of the RoB tool. One unexpected value of the RoB tool is in getting inexperienced reviewer to think about and understand the various sources of bias. However, it does slow reviewing down.

Appendix 2 - Risk of Bias Tool Evaluation Survey for Authors: Non-users

Detailed Results

Number of respondents: 132
Launch date: 01 Feb 2010
Close date: 22 Feb 2010

Section 1: Part A: Use of Risk of Bias tool

**1.** Please give a reason(s) why you have not used the risk of bias tool			
I have not conducted a Cochrane review since introduction of the Risk of Bias tool:		n/a	95
I prefer a different method for assessing risk of bias or methodological quality:		n/a	8
The Risk of Bias tool complicates the process of making bias assessments:		n/a	4
Risk of Bias tool assessments are too time consuming:		n/a	2
**Other *(please specify)*:**			
• Did not know about it / Not heard about it			6
• A co-author was in charge of ROB			4
• Currently at the protocol stage of the review			4
• Not at that stage of the review yet			3
• Don't know how / still learning how to use it / prefer to get training first			3
• The remaining individual free-text answers (give below in full):			9
I am a member of the review group; I simply support authors to conduct the reviews. I have edited the Risk of bias entries however.			
I used some elements of the risk of bias tool.			
I don't know what the risk of bias tool is - do you mean the process of assessing bias in a Cochrane review (with tables etc in the handbook and on revman)? If so, I have used it, if not I have not.... so don't know what to do with this survey! sorry			
I find the risk of bias response options inadequate. I prefer an option that indicates something was done to some extent (i.e. medium risk of bias) rather than a binary option. And also I prefer to separate those studies which didn't do something because it wasn't appropriate from those who didn't do something or it was unclear and that this may introduce bias. May be useful to incorporate something about implementation and variation in implementation of the intervention- this is of particular importance in complex interventions and for public health but also of relevance to clinical topics. Failure to recognise this as a potential bias can lead to either overestimates or underestimates of effects.			
I and a co-reviewer have updated a review for a group which does not require use of the tool for updates.			
I have updated Cochrane reviews that did not previously use Risk of Bias tool. I am now working on a HTA systematic review that includes use of the EPHPP quality assessment tool.			
I am conducting a SRDTA and am unsure whether this is relevant			
We could not use it because we also included observational studies - and the RevMan software does not allow to activate or inactive the tool items for individual studies.			

Section 2: Part B: Training related to the Risk of Bias tool (non-users)

**2.** What type of training relating to the Risk of Bias tool have you participated in?			
I have attended at least one workshop at a symposium or colloquium:		n/a	14
I have attended at least one workshop independent of Cochrane symposia or colloquia:		n/a	7
I have attended Cochrane's standard author training:		n/a	15
I have read relevant training materials on my own time:		n/a	34
I have not received any specific training:		n/a	84

**3.** Have you read the guidance in the Cochrane Handbook related to the Risk of Bias tool?			
No. Please skip to question 4.:		65.6%	84
Yes. If yes::		34.4%	44
**3.a.** Please specify if you have:			
Read Chapter 8 Assessing Risk of Bias in Included Studies:		n/a	27
Read the Cochrane Handbook (Part 2) from start to finish:		n/a	14
Used the Cochrane Handbook to look up specific issues related to risk of bias:		n/a	27
**3.b.** Is the level of detail in the Cochrane Handbook related to the Risk of Bias tool:			
Not detailed enough:		9.1%	4
An appropriate level of detail:		88.6%	39
Too detailed:		2.3%	1
**3.c.** Do you feel the provision of further examples of risk of bias assessments would be beneficial?			
No:		11.4%	5
Yes. If yes::		88.6%	39
**3.c.i.** Would you recommend:			
General examples:		n/a	13
Examples specific to each Cochrane review group:		n/a	18
Examples specific to types of interventions (e.g. drugs, behavioral interventions, complex interventions):		n/a	26
Other *(please specify)*:		n/a	3
Examples specific to study designs eg cluster randomised trials, crossover trials, and designs other than RCTs for reviews that included these.			
I would like to see more about observational studies. I would prefer the term "masking" over blinding. Why not, after all, if a health area uses "blind" as an outcome? I am not sure I agree with all the different types of bias you outline. It is really 2-selection and information.....			
Risk of bias may differ across studies.			

**4.** Have you received any guidance from a Cochrane review group related to the Risk of Bias tool?			
No. Please skip to question 5.:		84.4%	108
Yes. If yes::		15.6%	20
**4.a.** Please describe that guidance:			
I was advised to read Chapter 8 Assessing Risk of Bias in Included Studies in the Cochrane Handbook:		n/a	8
I was given specific written guidance developed by my review group:		n/a	1
I was given specific verbal guidance by my review group:		n/a	6
I was advised to enroll in a workshop related to the Risk of Bias tool:		n/a	2
Other *(please specify)*:		n/a	4
a co-reviewer indicated this was a new development and that it should be added to the protocol and data extraction forms			
given advice from my co authors who have used the ROB tool			
I received training at the editors meeting of the Cochrane Stroke Group			
There is a wealth of other literature on risk of bias including reading some Cochrane Reviews, Doug Altman's textbook and personal reflection on reviews where one knows the inside of the subject.			
**4.b.** Do you feel that the guidance provided by your review group was:			
Excellent:		10.0%	2
Very good:		45.0%	9
Good:		40.0%	8
Fair:		5.0%	1
Poor:		0.0%	0

**5.** Do you feel the availability of training materials for personal use (e.g., Handbook and other written guidance) related to the Risk of Bias tool is:			
Insufficient:		29.6%	34
Sufficient:		65.2%	75
More than sufficient in relation to other required training materials for other topics:		5.2%	6

**6.** Do you feel the avaliability of training events (e.g. workshops) for the Risk of Bias tool is:		
Insufficient:	44.5%	49
Sufficient:	50.0%	55
More than sufficient in relation to other required training events for other topics:	5.5%	6

**7.** What format of training for the Risk of Bias tool would you be **MOST** likely to access?		
Training that is incorporated into standard author training:	14.3%	18
Online training, including webinars:	58.7%	74
In-person workshop: ½ day:	9.5%	12
In-person workshop: Full day:	10.3%	13
In-person workshop: Two days:	2.4%	3
Other *(please specify)*:	4.8%	6
Any of them		
Either training that is incorporated into standard author training Online training, including webinars		
I would have to decide after looking first at the Handbook Description. Probably an online training session, or help from my own Cochrane Review Group.		
I'd prefer a half- or one-day workshop, depending on where it was, backed up by online training (I don't know what a webinar is - it looks real-time?- the instructions would have to be really good for me to attempt to use an unfamiliar on-line format for training - it feels like too much to learn at once!)		
No training should be needed if it is a useful tool		
Tell me about it and I'll use it as I use rev man, using help screens as appropriate		

**8.** What level of training would you be **MOST** likely to access:		
Beginning:	48.4%	60
Intermediate:	36.3%	45
Advanced:	15.3%	19

**9.** If you have any other comments related to the Risk of Bias tool that you would like to make, please record them here.
**Re: Training related comments**
More training session could give authors an insight of how to use the risk of bias tool.
re. question 8 *[level of training]*, I would start at the beginning with the aim of being able to do them all.
Hands on training is preferred.
The use of a self-guided web-based training would most likely be the most accessible type as well as one that I may be able to squeeze into life.
Simple and Rapid Guidelines Handbook is needed.
I believe can be a little easier if it is done along a review which will serve as practicals
**Re: RevMan related comments**
I like the graphs!
The functionality of the RevMan software was the problem, not the risk of bias assessment itself.
I published a Cochrane review in 2003 with an update in 2009 but was not aware of this tool. I will make a point to check this out before the next update. Maybe it would help if the RevMan software requested that authors use the tool, with an option to decline if the authors prefer another approach.
My colleagues use the RoB tool and it seems to cause no problems, to provide a good summary of the quality of the included studies. I like the colourful summary graphic.
**Re: Other comments**
Guidance from experienced methodologists should be provided throughout the review process on topics or specific situations that may be difficult to address and the support should be regarded as mandatory by the Cochrane Group .
I prefer to use a risk of bias approach that is proposed by PEDro as the decision rules are unambiguous
Would be interesting to collect specific risk of bias tools for each type of intervention. Also to see the extent to which risk of bias can be controlled across different type of interventions. While blinding of care provider is possible for pharmacological interventions, it is less for psychological interventions.
I think I will be in a better position to comment once I have completed an update of one of my existing reviews
I have not read any of the Cochrane manual info on Risk of Bias tools so I was unable to answer those questions. However, I am very interested in the tool and want to learn more about it.

Appendix 3 - Risk of Bias Evaluation Survey: Editors & CRG staff

Detailed Results

Survey Overview

Number of respondents: 58
Launch date: 01 Feb 2010
Close date: 22 Feb 2010

The results from the survey are presented below question by question.

Section 1: Part A: Descriptive Information

1. What is your role within the Cochrane Collaboration? (select one)

		% out of 58	Responses
Managing Editor:		32.8%	19
Coordinating Editor:		19.0%	11
Editor:		19.0%	11
Trials Search Coordinator:		3.4%	2
**Other *(please specify)*:**			
• Assistant Managing Editor		1.7 %	1
• Centre staff		1.7 %	1
• Centre trainer		1.7 %	1
• Methodologist		1.7 %	1
• Author (of review / protocol) *		19.0%	11

* Eleven authors filled in the survey aimed at CRG Staff despite being asked to complete the survey aimed at authors.

Section 2: Part B: Use of the Risk of Bias Tool

2. Which statement best describes how your review group implements risk of bias assessments, using the Cochrane Risk of Bias tool, in **NEW** Cochrane reviews?

We require that all new Cochrane reviews include risk of bias assessments:		77.6%	45
We recommend, but do not require, that all new Cochrane reviews include risk of bias assessments:		15.5%	9
We do not have a policy on risk of bias assessments in new Cochrane reviews:		1.7%	1
I am not sure / I do not know:		5.2%	3

**3.** Which statement best describes how your review group implements risk of bias assessments, using the Cochrane Risk of Bias tool, in **UPDATED** Cochrane reviews?		
We require that all updated reviews include risk of bias assessments:	48.3%	28
We recommend, but do not require, that all updated reviews include risk of bias assessments:	37.9%	22
We do not have a policy on risk of bias assessments within updated reviews (Please skip to question 4):	5.2%	3
I am not sure. (Please skip to question 4):	8.6%	5
**3.a.** For updates to Cochrane reviews, does your review group policy/guidance apply to:		
Only new studies included in the update:	9.7%	3
Both new and original studies included in the review:	77.4%	24
I am not sure:	12.9%	4

**4.** Does your review group verify, in any way, the risk of bias assessments that are made within Cochrane reviews submitted to your group?		
Unsure:	13.8%	8
No:	32.8%	19
Yes. If YES,:	53.4%	31
**4.a.** Does your verification include?		
Assessments against reports of included studies:	n/a	18
Assurance from peer reviewers that assessments are reasonable:	n/a	10
Ensuring that selected quotations from reports of included studies have been entered correctly:	n/a	7
Other *(please specify)*:	n/a	13
Comparison with our assessments as authors of other reviews with studies in common		
Cross check rating with quotation and check against original article where there is doubt.		
Depending on the experience of the reviewers, I check a sample of the assessment of included studies, on main items only.		
may involve checking study reports if deemed necessary		
on a small random sample		
Quality advisor check the assessment		
Sometimes via assessment against reports of included studies. Depends on author team and/or resources available.		
the verification can be several stages - starting with ensuring the quotations agree with the yes/no/unclear judgement. If there are items which cause concern we would refer to full papers in those cases.		

There is an independent validity team that assesses all studies. There are explicit decision rules for interpretation that include contextualized (i.e. treatment specific) issues. The validity team ensures consistency across reviews and includes clinicians, methodologists, and a statistician.
Verification is up to the judgement of individual editors but generally at least one article will be checked and there are also checks for internal consistency. Thus if clearly a quasi-RCT, we wouldn't expect allocation concealment to be Yes.
We are not able to check all ROB tables but do verify some if we are in doubt about the content.
we do not do this for all reviews but have done it for some where I think this has not been done properly
we have not yet set out a systematized way of checking; it is more ad hoc. e.g. if an assessment indicates that a trial has a low risk of bias, but other assessments have indicated otherwise in past reviews; if the text, table of included studies and ROB table are inconsistent; if the peer referees question the assessment based on their knowledge of the trials

**5.** Does your review group recommend that authors pilot test (for example, initial inter-rater testing, or testing decision rules) their process for making risk of bias assessments?			
Unsure:		13.8%	8
No:		51.7%	30
Yes. If YES,:		34.5%	20
**5.a.** Does your review group recommend?			
Assessments by more than one reviewer:		n/a	19
Modifications to the approach suggested in the Cochrane Handbook:		n/a	2
Explanation or elaboration of the approach suggested in the Cochrane Handbook or by your review group:		n/a	6
Other *(please specify)*:		n/a	5
I grow concerned about the 'blinding' category when the intervention is psychological or exercise, etc (i.e., where blinding is only possible for outcome assessors) that authors understand that they still need to consider these risks of bias			
Not exactly modifications, but we usually suggest for new reviews (and updates) to look for bias associated with major baseline imbalance and lack of care programme comparability (especially in terms of care provider expertise and rehab)			
our group expects that all review groups pilot test the assessment tool - whether they do or not ??? we can only go by what they write			
our guidance is that all stages of the data extraction and risk of bias assessment process be undertaken by at least 2 review authors independently			
we do not require the calculation of any agreement statistics, however			

**6.** Does your review group recommend use of the Cochrane Risk of Bias tool to assess the risk of bias for designs **other than randomized trials**?			
Unsure:		26.3%	15
No:		45.6%	26
Yes. If YES,:		28.1%	16
**6.a.** Does your review group recommend use of a **modified** Cochrane Risk of Bias tool for this purpose?			
Unsure.:		0.0%	0
No.:		31.2%	5
Yes:		68.8%	11
**6.a.i.** If yes, please specify how the Risk of Bias tool was modified			
Added new bias domains (e.g., a new domain about the similarity of treatment groups at baseline):		n/a	10
Deleted existing bias domains (e.g., sequence generation, blinding):		n/a	5
Modified criteria from the Cochrane Handbook for making judgments about bias domains:		n/a	5
Other *(please specify)*:		n/a	4
depending on study type according to criteria derived from EQUATOR guidance			
I have clicked yes above in order to answer the subsequent qqs. This is work in progress, NOT set out in current Handbook.			
we have used a blended list of criteria in reviews that include both RCTs and cohort studies (with a comparison group)- we use the NOS, as per the Handbook (even though the NOS wasn't really developed for intervention studies but for prevalence studies)+ the Cochrane ROB + the additional items that have been deemed important for trials in our field -operational definitions for all criteria are included in an appendix as part of our standards			
We try to give advice in accordance with the Handbook for assessing non-randomised studies, although the advice in the Handbook is not complete.			
**6.b.** Please specify for what study design(s) your review group recommends use of a Cochrane Risk of Bias tool			
Quasi-randomized studies:		n/a	15
Cohort studies:		n/a	8
Case-control studies:		n/a	5
Interrupted time-series:		n/a	7
Controlled before and after studies:		n/a	10
Other *(please specify)*:		n/a	4
+ of course the RCTs - these are the only study designs that are accepted to date in our reviews - this does not refer to the DTA reviews, only intervention reviews			
Any study that is included in the review, in accordance with the Handbook and the CRG's advice. Have not considered using it for qualitative, health economics, etc.			
Our Review Group is atypical in the types of study and the types of evidence it reviews.			
We don't explicitly recommend study designs			

**7.** Does your review group recommend use of a **modified** Cochrane Risk of Bias tool to make assessments of bias of **randomized trials**?			
Unsure:		14.0%	8
No:		63.2%	36
Yes. If YES,:		22.8%	13
**7.a.** Please specify how the Cochrane Risk of Bias tool was modified			
Added new bias domains (e.g., a new domain about the similarity of treatment groups at baseline):		n/a	10
Deleted existing bias domains (e.g., sequence generation, blinding):		n/a	4
Modified criteria from the Cochrane Handbook for making judgments about bias domains:		n/a	3
Other *(please specify)*:		n/a	2
I'm not sure about this question. When discussing the 'setting up' of tables, I frequently engage with authors to ask what they regard as especially important risks of bias. For some with, say, manualised education interventions, implementation integrity. For an author who is willing to accept crossover data, clarity on washout might be important. Etc.			
Modifications largely focus on application of current domains to cluster randomized trials			

**8.** How does your review group recommend that authors report risk of bias assessments in Cochrane review(s)?			
Complete the risk of bias tables:		n/a	48
Include one figure(s):		n/a	18
Include a figure(s) and a table:		n/a	19
I am not sure:		n/a	6
Other *(please specify)*:		n/a	10
also include a summary of the methodological quality in the review			
Figures not routinely recommended.			
in addition we encourage authors to draw on the RoB assessment within the text of the results when presenting data from trials and making judgements on the strength of the evidence			
Recommendation of inclusion of a figure or a table is made on per review, we do not apply a general 'rule'.			
See above, work in progress.			
There are 2 figures that can be activated to show RoB visually (summary and by individual trial) - this looks good			
Using a figure can be helpful but is optional.			
we also make the headings in the results section mandatory			
We consider the figures optional.			
We don't specify whether figures are a good idea or not. I do try to look and say if a figure might be misleading esp. if for example one trial is bigger than all the others, which makes the bar figure misleading in some ways... (as the real risk of bias isn't 'weighted'			

9. How does your review group recommend that authors incorporate risk of bias assessments into a meta-analysis and/or conclusions of Cochrane review(s)?

We recommend that authors conduct sensitivity analyses by risk of bias:	n/a	33
We recommend that authors restrict the primary analysis to studies at low risk of bias:	n/a	5
We recommend that authors include a summary within the interpretation of results:	n/a	24
We do not make any specific recommendations regarding the incorporation of risk of bias assessments into meta-analyses or conclusions of the review:	n/a	15
Other *(please specify)*:	n/a	7

at least for allocation concealment
I have not found any way of using it except in very general terms to say that 'most of the trials were at some risk of bias' We rarely have enough trials to select the better ones on any criteria
No idea
Our reviews certainly include all of the above. I am not sure whether we have a specific policy though
we encourage authors to drawn on the RoB assessment within the text of the results when presenting data from trials and making judgements on the strength of the evidence. Some authors do conduct sensitivity analyses - and this is encouraged whilst not being a formal group policy
We recommend that authors consider all these options, in accordance with the Handbook, but do not give absolute recommendations due to the diversity of reviews and CRGs we interact with.
We're not at this stage yet.

10. Does your review group recommend that quotes (cut and paste) from included studies are included to support risk of bias assessments in risk of bias tables ?

Unsure:	24.1%	14
No:	17.2%	10
Yes:	58.6%	34

11. Do you think the request for quotes to support risk of bias assessments:

Adds transparency to risk of bias assessments:	n/a	49
Increases confidence in risk of bias assessments:	n/a	30
Adds little value to the risk of bias assessment process:	n/a	9
Unnecessarily adds time to the risk of bias assessment process:	n/a	5
Other *(please specify)*:	n/a	8

Although, entire quotes are not added, we do on occasion also reference the page number
But if used obsessively increases the size of tables even more. Worth using selectively
I am strongly in favour of quotes. I can stand disagreeing with someone over their judgement. I can't stand having no idea how they arrived there.

I realize that this may sound confusing, but at this point, for example, sequence generation is mentioned in the table of included studies, and again in the ROB table and sometimes in the text, making it a little repetitive and confusing for authors; ditto for some of the other criteria
I'm pretty ambivalent about the RoB. It clearly adds transparency and enables better judgement on the author's performance (often disappointing) but it is much more involved from everyone's perspective (and it certainly holding up delivery of protocols - often a key problem area - reviews and updates up). Sometimes the benefits are not obvious even though theoretically RoB is sounder.
It allows anyone else to check the authors' judgement so improves reliability
We don't use quotes
We encourage use of text in any assessed domain, to ensure it doesn't get suppressed on publication as a 'blank' field.

Section 3: Part C: Your opinions about the Risk of Bias tool

**12.** Overall, compared to RevMan's past practice of recording only allocation concealment, the current recommended practice of assessing risk of bias is:			
Much better:		65.5%	38
Somewhat better:		29.3%	17
About the same:		0.0%	0
Somewhat worse:		0.0%	0
Much worse:		0.0%	0
I am unsure:		5.2%	3

**13.** Please specify aspects of the Risk of Bias tool that you **DO** like			
Ability to provide information (e.g., quotes from reports) to support a judgment:		n/a	48
Flexibility:		n/a	19
Good framework:		n/a	32
Standardized approach:		n/a	46
Table(s):		n/a	31
Figure(s):		n/a	33
Can be completed quickly:		n/a	11
There are no aspects of the Risk of Bias tool that I do like:		n/a	2
Other *(please specify)*:		n/a	5
It seems to make review authors critically appraise studies more thoroughly and as such this is more reliable than a tick box exercise for quality scales.			
The act of engaging with ROB in this way increases the likelihood that it will be taken account of in interpreting results and drawing conclusions.			
The classification to "yes" and "no" is unclear. Blinding has more than 2 states			
Thoroughness of the assessment, and detailed guidance in implementation.			
We have been requiring authors to include their assessment of 11 criteria viz internal validity in an additional table since 1997 - the figures are an improvement! The downside is that many authors still can't figure out how to activate/add criteria, so this is something that is better done in the editorial office. But, the additional tables weren't always so pretty either:)			

**14.** Please specify aspects of the Risk of Bias tool that you **DO NOT** like			
Time taken to complete assessments:		n/a	20
Table(s):		n/a	2
Figure(s):		n/a	5
Increased complexity:		n/a	18
Assessments are too subjective:		n/a	16
Request for quotes:		n/a	2
Difficult to modify:		n/a	8
The meaning of "Yes/Unclear/No" assessments is unclear:		n/a	24
There are no aspects of the Risk of Bias tool that I do not like:		n/a	9
Other *(please specify)*:		n/a	18

**Re: General comments**
I have not to date (because of staff shortages) checked the RoB but think in future this design will make it easier to check.
Over simplified
**Re: Unclear language**
Currently ambiguous. I would like the responses to be either: High/Unclear/Low (risk of bias) or the meaning to be reversed so that Yes means ROB (criterion not met) and No means Low ROB.
For those whose first language isn't English, the RoB wording is definitely a problem. But also the way of wording means that even native English speakers have to concentrate. I've lapsed once or twice! It is a right pain to add in the quotations.
Section of the responder's answer that was placed under another subsection but contains a quote relevant to this subsection, so copied here: "…I would prefer low/high/not adequate reported - but, even better, a 5 point scale…"
I do not like the translation of yes/no to low and high risk of bias. I find the explanation of 'Incomplete outcome data addressed?' too complicated, and would narrow it down to 'Intention to treat analysis performed?'.
**Re: Difficulties in making risk of bias judgements**
Assessments depend on expertise and experience which reviewers lack
In many trial reports the information is unclear. -Surprisingly hard to take a consistent approach either across trials or when revisiting the same trial. -Handbook advice on wording seems very 'clunky' -Unclear that it adds anything to the strength of the review conclusions.
I don't think its a matter of not liking them, but it does take some authors more time than others to 'grasp' the nuances and subtleties of some of the criteria - I'm not sure that is new, perhaps it is just more apparent with the new format.
I worry about the judgements made by some reviewers - important to get agreement data for double assessment by 'typical' reviewers. I would prefer low/high/not adequate reported - but, even better, a 5 point scale. I worry about the complexity of some judgements (e.g. by lumping all aspects of blinding/obj outcome together). For all that quoting from the paper adds transparency, I think judgements could go horribly wrong (given previous experience with allocation concealment).
Really have to be clear that I can't believe you have a choice of 'assessments are too subjective'. Assessments ALWAYS were subjective. For the first time now we have the chance to see how authors actually translated the

quotes into the judgements. Particularly informative when updating.
**Re: Including ROB assessments into the meta-analysis /review conclusions**
It is unclear from the Handbook what review authors should do with the information and summative judgements are difficult to make when the bias tool has broken down bias by potential source. Conceptually it is also quite hard to remember that judgements are as much about how well the study addresses the review question, as it is about how well the study was designed & performed.
It takes time, but one wonders about the consequences of the time not being taken. My major concern is authors who complete a RoB table but do not then use it, without prompting that is.
**Re: Tables and figures in RevMan**
Risk of bias tool should not be attached to the 'Characteristics of included studies table' but be a (table) structure in itself
The summary graph is useless when the sizes of the studies vary.
It is not obvious to authors that there are more than one assessments available in the RoB tables, as it isn't obvious they need to click on the cog wheel to see all the assessments. We end up having to explain how to do this to every set of authors.
**Re: Specific domains issues**
Complicated to include multiple outcomes data.
Section of the responder's answer that was placed under another subsection but contains a quote relevant to this subsection, so copied here: "… I worry about the complexity of some judgements (e.g. by lumping all aspects of blinding/obj outcome together)… "
when 'other sources' of bias are added care must be taken by the author in asking the question to ensure the yes/no is correctly coded. Need more guidance on 'other sources' authors often add inappropriate criteria.
**Re: Application to non-RCTs**
Could use more advice on how to implement for non-randomised studies. Variations in implementation between CRGs undermine the system and confuse authors and trainers. Writing overall assessments is difficult, but I don't think this is something that needs to be fixed.

**15.** Do you have any suggestions to make the Risk of Bias tool easier to implement in Cochrane reviews?
**Re: General comments**
I would like to see research evidence that it is actually useful in modifying the process or conclusions of a review. It seems like a complex and subjective task that does not provide any information that is useful in interpreting the review.
It is hard to make this easier (see above).
"…Needs training and more training for authors…"
Provide more examples.
"… Consistent application across CRGs…"
Keep insisting and require a proper statistician in the group
Opportunity to explicitly state how some decisions were operationalized (to assist readers in understanding ratings). This should be stated once...and be accessible to readers.
**Re: Unclear language**
"…need revision of Yes = low risk of bias, No = high risk of bias …"
need to clarify the difference between unclear and not reported and inadequate and how these assessments impact on potential bias. Needs training and more training for authors
Alas, I think my concerns/suggestions above will probably make the tool more difficult rather than easier. Clarifying yes/no/unclear would help! 5-point scale may (paradoxically) remove some agonising about yes/no

decisions. Making unclear=not adequately reported may stop reviewers using unclear when they can't decide yes/no (does this happen?).
Some rewording may help. The lack of appearance of unclear in the Figures if no text available perhaps should be reconsidered.
Reword 'Yes' 'No' or 'Unclear' to 'High', 'low' and 'unclear' risk of bias; provide examples from existing Cochrane reviews in the Handbook that show why judgements were made for each item.
**Re: Difficulties in making risk of bias judgements**
"…provide examples from existing Cochrane reviews in the Handbook that show why judgements were made for each item…."
"…Bank of good examples of RoB tables and RoB assessments in the text of a review…"
Would like a 'Not applicable' option as well as the Yes/No/Unclear
The table which gives guidance on making judgements (in the handbook) is very useful. However I have come across many people who have done reviews and didn't know that this table existed. It should be made more prominent.
"…2. Despite the improved (greater) level of detail given in the Handbook, judgement as to Risk of Bias still seems quite subjective and at risk of diverse assessments by different authors. …"
**Re: Tables and figures in RevMan**
A pop up of the definitions and examples for each criterion would be great!
Need revision of Yes = low risk of bias, No = high risk of bias. Would it be easier if ALL domains were selected initially by default and the author had to deselect the ones they choose not to use (??)
I find it very difficult to switch it on and build the table in the first place. The blinding (of all outcomes...) is particularly difficult to work. Really, please make it physically easier to create....
**Re: Specific domains issues**
I understand why the criteria for blinding and missing data have been grouped, but, in keeping with my response to Q14, I think less experienced authors do not think that there are three aspects of blinding that should be addressed and two aspects of missing data, which is why we continue to break them out into separate criteria. if an experienced review team addresses all the aspects in a single item, we will leave it alone, but in a field where blinding of providers and subjects is usually impossible, it is important to keep it in their face that just because they can't blind, doesn't mean that bias isn't introduced. there are also different attempts being suggested by methodologists that may be considered a proxy to blinding that we have incorporated into our operational definitions
Restrict incomplete outcome data and blinding to primary review outcomes
Blinding and missing data components are a bit 'clunky' at present. Need clearer instructions about selective outcome reporting - and make more practical - very few trials publish their protocols, for example
**Re: Application to non-RCTs**
1. We need standardised authoritative (ie. Cochrane Handbook) in depth guidance on applying the ROB tool to quasi-random, CBA and ITS study designs. 2. Despite the improved (greater) level of detail given in the Handbook, judgement as to Risk of Bias still seems quite subjective and at risk of diverse assessments by different authors.
More advice for implementation in non-randomised studies. Bank of good examples of RoB tables and RoB assessments in the text of a review. Consistent application across CRGs.

**16.** Based on your experience with your review group, do authors regularly encounter problems making assessments within the sequence generation domain?			
Unsure.:		19.0%	11
No.:		51.7%	30
Yes. If YES,:		29.3%	17
**16.a.** Are the problems related to:			
Consistency between assessors:		n/a	2
Confusing sequence generation with allocation concealment:		n/a	13
Difficulty in assessing whether a particular method was associated with bias:		n/a	8
Adequately distinguishing between different non-random allocation processes:		n/a	4
Other *(please specify)*:		n/a	2
I'm not sure it's 'regularly', but inexperienced authors don't always 'question' the validity of the sequence generation in trials that just say the participants were 'randomized' without giving details, so sometimes you get a 'yes' without verification other than the quote of 'randomized'			
Mostly fine, but if a complex, 'statistical' description, is found, it can be difficult for non-statisticians to interpret.			

**17.** Based on your experience with your review group, do authors regularly encounter problems making assessments within the allocation concealment domain?			
Unsure.:		20.7%	12
No.:		29.3%	17
Yes. If YES,:		50.0%	29
**17.a.** Are the problems related to:			
Consistency between assessors:		n/a	7
Difficulty in assessing whether a particular method was associated with bias:		n/a	13
Confusing allocation concealment with blinding:		n/a	21
Other *(please specify)*:		n/a	2
It's depressing but even before RoB reviewers were making wrong calls - nothing changed then.			
Poor reporting leading to difficulty classifying ROB. Also what to do where report merely states "double blind;" and whether this sufficient to conclude allocation concealment.			

**18.** Based on your experience with your review group, do authors regularly encounter problems making assessments within the blinding domain?			
Unsure.:		12.5%	7
No.:		28.6%	16
Yes. If YES,:		58.9%	33
**18.a.** Are the problems related to:			
Consistency between assessors:		n/a	7
Confusing blinding with allocation concealment:		n/a	15
Difficulty in making a global assessment of blinding of patients, providers and outcome assessors:		n/a	27
Difficulty distinguishing between double and triple blind:		n/a	9
Making an assessment for blinding when patients and/or providers cannot be blinded:		n/a	19
Other *(please specify)*:		n/a	9
Especially when it is not possible to blind the patients or providers, it is not appropriate to use this as a marker of 'bad' quality			
I have addressed this in previous questions (Q15), but in addition to what I have already stated, the use of the term 'double' and 'triple' blinding means different things to different people so is generally not accepted by our group without definition; allocation concealment is sometimes taken as blinding;			
I have an author who is very angry at being asked to discuss blinding for an intervention that can't be blinded (for patients and providers). Our statistician believes (rightly in my view) that it's particularly important as in *[our clinical area]* outcomes are often self-assessed, so blinding is effectively impossible across the board. The author simply thinks we're being 'hard' on investigators who couldn't run trials any other way: we think *[the author]* can't grasp that *[he/she]* simply has to take on board that with the best will in the world (and the best investigators) the fact that these trials are genuinely at a higher risk of bias than placebo-controlled trials of other interventions is, sadly, an inevitable fact. Not an 'insult'. This must come from the fact that authors used to concepts of 'methodological quality' can't move on to 'risk of bias' because they think we're still 'dissing' quality instead of describing bias in these cases.			
Judging if blinding of, for example, the patient was adequately blinded. The judgement of adequate is difficult for many reviewers.			
Making the additional judgement about whether lack of successful blinding is likely to have impacted on results.			
The separation by outcome is an issue here too.			
We partition out blinding into the three main categories of provider, patient and outcome assessor.			
we tend to encourage our authors to consider blinding of patients, providers and outcome assessors separately - or concentrate on blinding outcome assessors			
when trials are described as double blind (without detailed explanations) it is not always clear whether it is the patient and outcome assessor who are blinded or the patient and clinical care provider			

**19.** Based on your experience with your review group, do authors regularly encounter problems making assessments within the incomplete outcome data domain?			
Unsure.:		17.5%	10
No.:		10.5%	6
Yes. If YES,:		71.9%	41
**19.a.** Are the problems related to:			
Consistency between assessors:		n/a	11
Overall complexity of guidance:		n/a	13
Difficulty in making an assessment when the drop out rate is described but not acceptable:		n/a	31
Difficulty establishing whether an intention-to-treat analysis has been completed:		n/a	33
Difficulty establishing what constitutes "complete" outcome data:		n/a	27
Difficulty making assessments of missing outcome data at different follow up periods:		n/a	29
Confusing incomplete outcome data with selective outcome reporting:		n/a	17
Other *(please specify)*:		n/a	8
Again, the different outcomes adds to the complexity.			
and different interpretations of what constitutes ITT - the most important one (participants remaining in their allocated groups) does not seem to figure in the RoB assessment			
difficulty in applying this bias domain to several types of outcomes			
Difficulty with the idea of having to calculate the potential impact on each result of missing participants, especially continuous data.			
Impossible to do for all review outcomes			
We have set some internal decision rules to judge these situations. However, it is not clear if our approach is used by others.			
When appraising survival analysis, the LTF is complex to calculate and may indeed not be as critical. This involved fairly high level statistical insights			

**20.** Based on your experience with your review group, do authors regularly encounter problems making assessments within the selective outcome reporting domain?			
Unsure.:		14.0%	8
No.:		19.3%	11
Yes. If YES,:		66.7%	38
**20.a.** Are the problems related to:			
Consistency between assessors:		n/a	8
Difficulty making an assessment without access to a study protocol:		n/a	33
Confusing selective outcome reporting with incomplete outcome data:		n/a	17
There being no standard means of measuring outcomes in your field:		n/a	14
Other *(please specify)*:		n/a	9
Generally a trial only reports a few outcomes anyway so there is no way of getting data for outcomes that should have been collected but were not.			
in the definitions given in the handout, one of the criteria for the judgment of 'no' = 'one or more outcomes of interest in the review are reported incompletely so that they cannot be entered in a meta-analysis' => to confusion as to what bias this creates			
It is not really clear what this domain means for our reviews and we should probably tell authors not to use it.			
need to get protocol from clinicaltrials.gov or similar. Authors seem to prefer not to do this as too much additional work			
Resistance to the idea of chasing down the study protocol, and feeling it's somehow harsh to make an 'unclear' judgement without one, as they are so rarely available (as the 'unclear' judgement is considered negative).			
Some authors view this as 'unless you catch them out, every trialist gets a Yes'. Others are more circumspect and give everything an 'Unclear'. Hard to convince the 'optimists' (see as above for blinding, and for some of the same reasons - authors just unwilling to appear to call investigators into question, as they see it, particularly if there is less of a tradition of publishing trial protocols eg with psychological interventions than pharma ones).			
this is routinely based on the little information available in the publication			
We have set some internal decision rules to judge these situations. However, it is not clear if our approach is used by others.			
We tend to find that the outcomes we want are reported as part of a 'composite outcome' in the study. Does this introduce bias if we cannot get the data in our review?			

**21.** Based on your experience with your review group, do authors regularly encounter problems making assessments within the Other sources of bias domain?			
Unsure.:		19.3%	11
No.:		24.6%	14
Yes. If YES,:		56.1%	32
**21.a.** Are the problems related to:			
Consistency between assessors:		n/a	5
Difficulty determining what other types of bias to consider:		n/a	29
Other *(please specify)*:		n/a	6
As explained above we suggest 2 other domains, but it can be quite enough to stick to the core set of domains.			
early stopping causes problems and should be added in here.			
Hard to know whether to fill in "unclear" or "yes" (since other potential sources of bias may b unknown)			
Occasionally things we would not consider to be bias			
Often want to report sample size, other non-bias issues.			
Within our group there are some variations as to adding "other" items dependent on the type of intervention. For example, funder bias may be operationalized slightly differently within our group as the central validity team does not rate this item.			

**22.** Do you think the Other sources of bias domain is helpful to make Risk of Bias assessments			
Unsure:		29.3%	17
No:		24.1%	14
Yes:		46.6%	27

**22.a.** Please explain your response:
**Those who answered UNSURE:**
Depends how it is used - as a collective for exceptional bias (major baseline discrepancies; contamination). So it can be but difficult to know how to incorporate it into an overall assessment.
For most reviews it is not obvious what this might be used for - possibly fro reviews including non - random studies
It is not clear what should be considered in this domain.
Most authors indicate no other sources of bias, but probably because they lack guidance in thinking of any other potentially relevant sources.
Not consistent across reviews; most authors just choose, 'yes' free from other bias; it is too vague
**Those who answered NO:**
being explicit about the bias would be better
I prefer that reviewers add a specific item to the tool, rather than have an item 'other'
I think most of the domains are difficult enough without having to think of any more
It is easy to adapt the tool to add a specific bias. A general assessment is not useful, because to subjective
We tend not to use it.
**Those who answered YES:**
Adds flexibility
Allows authors to indicate when trials are industry-sponsored or to highlight an issue that may have occurred in a particular trial that is not very usual.
Essential for alternative designs. Further some ROB are likely context specific
for authors who know what they are doing
for early stopping it is critical as this is not captured elsewhere
for example for adding domains of importance in the assessments of cluster trials
gives people an option but they often don't fill it
It allows flexibility
It allows for factors not covered in the other domains
it helps to capture those things that may be specific to a study, a review, or a particular topic
it is a good place to flag an issue that could be significant even if it is just rated unclear
It is helpful, because it adds flexibility, but I appreciate that some authors find it redundant often.
it would be good to have more guidance on acceptable ' other sources' and also perhaps be able to check with the methods group on some review group regulars to ensure they are suitable
May need clarification but helpful to be able to record issues that are either specific to research question or specific to a particular paper ('smell a rat' issues)
Only place you can assess funding bias
Small studies with (chance?) baseline imbalance for prognostic variables common in our area; it is important for us to flag this.

There are clearly some additional issues that are useful to report when they occur. Having the 'other' box is a good reminder to consider them.
This is very dependent on the type of intervention.
to add other important topics like funding
To capture the sources of bias that impact on specific types of intervention/study.
while the 5 main criteria are obviously important for assessing internal validity, they are not the only potential risks of bias that may affect our confidence in the results. Arguably, if only highly skilled methodologists, statisticians and clinical researchers conducted Cochrane Reviews, these five would tell one quickly if a trial had a high/low risk of bias. However, since the implications of some of these criteria may not be readily apparent to less experienced authors (who likely form the bulk of our authors) it is important to address other issues in this important assessment.
Yes. It allows you to record things that in particular you may not have dreamt of at protocol stage. Again, say with a psychological intervention, finding out that for reasons of economy, the same teacher or therapist delivered both arms of the trial on different days. You can record possibility of contamination - which never occurred as a risk at protocol stage

**23.** Does your review group recommend that authors include **standard** "Other sources of bias" in Cochrane reviews?			
Unsure.:		29.3%	17
No.:		53.4%	31
Yes. If YES,:		17.2%	10
**23.a.** Please specify what other sources of bias:			
As above: baseline imbalance; performance bias			
Depends on the trial			
For ITS designs (see EPOC criteria)			
Funding source			
source of funding			
I may misunderstand this question. We expect authors to consider whether or not there are other sources of bias i.e. we expect them to state that there are none (if that is the case).			
Mostly stick to the **12 items**, but some within our group add one or two items.			
these criteria have been identified and widely accepted as important in our field for many years (i) similarity at baseline (ii) avoidance or similarity of co-interventions (iii)acceptability of compliance (iv)similarity of timing of outcome measurement			
we did use - comparability at baseline / trial sponsorship by manufacturer - but have now reverted to standard guidance from Handbook - however authors often lob in 'others' which we then have to discuss with them.			
We include an additional ROW which suggests authors assess other quality indicators: • Were outcome measurement tools validated? • Are the outcome measures reliable? • Did the study obtain ethics approval?			

Section 4: Part D: Training related to the Risk of Bias tool

**24.** What type of training relating to the Risk of Bias tool have you participated in?			
I have attended at least one workshop at a symposium or colloquium:		n/a	29
I have attended at least one workshop independent of Cochrane symposia or colloquia:		n/a	12
I have attended Cochrane's standard author training:		n/a	6
I have read relevant training materials on my own time:		n/a	37
I have not received any specific training:		n/a	9

**25.** Have you read the guidance in the Cochrane Handbook related to the Risk of Bias tool?			
No:		5.2%	3
Yes. If YES,:		94.8%	55
**25.a.** Please specify if you have:			
Read Chapter 8 Assessing Risk of Bias in Included Studies:		n/a	44
Read the Cochrane Handbook (Part 2) from start to finish:		n/a	16
Used the Cochrane Handbook to look up specific issues related to risk of bias:		n/a	47
Other *(please specify)*:		n/a	3
haven't read all of part 2, but likely most of chapters 8-12			
How to make the 2 Figures appear			
Particularly the table ?8.5c			
**25.b.** Is the level of detail in the Cochrane Handbook related to the Risk of Bias tool:			
Not detailed enough:		18.2%	10
An appropriate level of detail:		76.4%	42
Too detailed:		5.5%	3
**25.c.** Do you feel the provision of further examples of risk of bias assessments would be beneficial?			
No:		12.7%	7
Yes. If YES,:		87.3%	48
**25.c.i.** Would you recommend:			
General examples:		n/a	22
Examples specific to each Cochrane review group:		n/a	14
Examples specific to types of interventions (e.g. drugs, behavioural interventions, complex interventions):		n/a	37
Other *(please specify)*:		n/a	7

...also show how the ROB impacted on the subsequent review analyses!
Actually I don't know - you cannot show every possible example and too many may confuse things further. I think ultimately a discussion must happen between authors and the Ed team if problems arise
each Cochrane group would be really nice, but it would have to be easily searchable so that folks could easily find what they are looking for. however, each group should have their own examples. at least giving examples covering different types of interventions would help -- also examples with only 1 or 2 small studies in which only partial data are provided. it is difficult for some groups to take examples that have the perfect huge trials to heart when they would never see trials like that in a whole career
Examples showing different methodological issues and how they have been assessed. A range of health topics would be useful, but not necessarily for every review group.
For me the Incomplete outcome data domain is the one that needs many more real examples of how to interpret.
I don't think the chapter should be cluttered up with more examples - but perhaps links to on-line examples could be provided? These could be real examples that have, in some way, been validated.
I learn better from examples than from theoretical text

**26.** Does your review group provide any guidance to authors related to the Risk of Bias tool?			
No.:		20.0%	11
Yes. If YES,:		80.0%	44
**26.a.** Please describe that guidance:			
We advise authors to read Chapter 8 Assessing Risk of Bias in Included Studies in the Cochrane Handbook:		n/a	41
We provide specific written guidance developed by our review group:		n/a	18
We provide specific verbal guidance:		n/a	13
We advise authors to enrol in a workshop related to the Risk of Bias tool:		n/a	3
Other *(please specify)*:		n/a	9
We also provide some examples from approved reviews			
we don't specify workshops because our authors come from such divergent areas; we do however advise them to consult cochrane.org for workshops that might be of interest/importance for them			
We edit their RoB tables, and help them if they contact us for help			
we give authors parts of the Handbook guidance as a word document which canb be attached to am email when asking them to complet a RoB assessment			
We give somewhat individualised advice, suggesting standard phrases, and to look at one of our other reviews. We also advise that they look at section in Handbook. We have been slightly inconsistent in advice on whether to include blinding or not			
We have a Study Quality Guide which is now in need of update, and a Data extraction Template utilising the RoB tool. We give authors feedback on their RoB assessments through detailed statistical editors' reports as well as peer review.			
We provide an example assessment form			
we provide standard protocol and review templates			
We WILL be modifying our RoB section in the Handbook once work in progress is complete.			

**27.** Do you feel the availability of training materials for personal use (e.g., Handbook and other written guidance) related to the Risk of Bias tool is:			
Insufficient:		31.6%	18
Sufficient:		63.2%	36
More than sufficient in relation to other required training materials for other topics:		5.3%	3

**28.** Do you feel the availability of training events (e.g. workshops) for the Risk of Bias tool is:			
Insufficient:		48.2%	27
Sufficient:		50.0%	28
More than sufficient in relation to other required training events for other topics:		1.8%	1

**29.** If you have any other comments related to the Risk of Bias tool that you would like to make, please record them here.
**Re: General comments**
I have serious reservations about the value of its use in making the conclusions of a Cochrane review more reliable. It is a lot of (difficult and tedious) work and I would like to be convinced of its benefit before either using it myself or insisting that others do so.
The question is whether the extra burden is worth it. It certainly has added to editorial burden (and length of RevMan printouts) and review authors are not happy with it regarding updates.
I think it is very useful to focus authors on components of validity and deter them from using methods that generate a total score
Great methodological advance that we strongly support.
**Re: Making RoB judgments**
[As an author as well as ME this response reflects both hats] Whilst I subscribe to the idea that bias is important and that it needs identifying, in practice I find it hard to be clear cut. The current table does make it possible to explain your judgement, but it is still easy to be, or appear to be, inconsistent. For example, allocation by shuffling a day's set of questionnaires seems on the face of it to be open to bias for both generation and concealment, but add in the context of a phone based trial with minimal contact between patient and provider, and no important difference in baseline characteristics, and my judgement of the likelihood of bias changes completely. (An example from a study that crops up in multiple reviews!) Generation and concealment are separate but also interact, Lots more examples in the Hanbook might help but could also reveal the lack of expert agreement.
**Re: Implementation of ROB judgements into review findings**
After completing ROB on each included study the author then has to write a "so what" summary in the results section. If all domains are yes or no that's not a problem - it is how to interpret the effect of unclear risk of bias in several domains. eg if all the studies are unclear in at least one domain, what can be said that is helpful for the reader of the review. Discussing the issues and their effects study by study is ok where Incl St are few but .....
**Re: Training**
I have responded 'insufficient' to qq. 27 and 28 *[on availability of training]* simply because I know some reviewers are struggling with the assessment. Hopefully this survey will highlight what extra training reviewers would like?
I haven't done the online distance training jointly developed by the UKCC and the University of Portsmouth yet, and would simply say I hope that a) it deals with ROB and b) it becomes more widely available (i.e., outside of the UK) very soon.
I indicated that training materials and events are currently sufficient - that is assuming that our authors are all experienced and able to tap into educational resources. we still need web-based resources for those who do not have the ability/resources to travel ... and maybe some way of testing their understanding

before the CRGs find out they don't have any?
Online training resources would be useful, is possible in multiple languages, for those authors unable to attend an in-person workshop, or who might wish to just have a friendly introduction to the system.
Some online training with lots of examples would be helpful,
worked examples would be good - working through trials with correct answers / explanations available. online training module in this would be good - again with plenty of examples

Appendix 4 - Risk of Bias Tool Evaluation - Focus Group Summary

Four focus groups were held to inform an evaluation of the Risk of Bias Tool (RoB tool). One focus group was held via teleconference on September 17, 2009 and three subsequent focus groups were held concurrently during the Cochrane Colloquium in Singapore on October 13, 2009.

The majority of focus group participants were experienced users of the RoB tool. Others were familiar with the RoB tool but had not yet used it in the context of a Cochrane review. The discussion focused on the RoB tool itself, as well as its associated guidance materials. Questions focused on experiences with the RoB tool, perceptions about the level of difficulty in using the tool and in summarizing RoB assessments at different levels, confidence in RoB assessments and perspectives regarding the sufficiency and adequacy of available training materials.

Following is a summary of the major themes that arose during the focus group discussion. The majority of themes outline problem areas, although suggestions for improvement were also provided.

Comparison to past practice

There was a fair amount of consensus among focus group participants that the RoB tool is an improvement over past practice; however, problems were identified that these individuals feel need to be addressed. Participants mentioned being content that there is now a standardized approach to bias assessments across review groups and that the RoB tool is more flexible than the prior practice of quality assessment, particularly due to the ability to add in new criteria particular to a review topic. In particular, the transparency provided by requesting quotes from study papers was identified as a strong improvement over prior practice, especially since the quotes are archived within reviews, which facilitates updates by different authors. Some feel the tool addresses more complex and relevant issues related to bias than previous assessments of study quality. The new guidance forces a different way of thinking (even related to previously assessed quality domains), which in some cases brings new insight to issues of study design.

Despite the overall positive appraisal of the RoB tool over past practice, a few participants expressed that the increased workload attributed to the new tool is a barrier to conducting reviews. Further, some participants expressed their perception that the distinction between past and current practice is not made clear enough and consequently some review authors continue to follow past practice and assess studies based on the best possible methods for a topic and not the risk of bias.

Positive aspects of the RoB tool

<u>Request for quotes</u>
The strongest benefit participants identified is that the RoB tool requests quotes from study papers to support reviewer assessments. Participants find this adds transparency to the review and adds confidence particularly for Managing Editors and peer reviewers, as it ensures clarity in how authors have made their assessments.

<u>Flexibility</u>

A few participants mentioned they appreciate the flexibility built into the RoB tool, for example that some criteria only need be applied at the outcome, versus study, level and that questions can be added within domains to make risk of bias questions more appropriate to a given review.

<u>Good framework</u>
Several participants indicated they appreciate the framework the RoB tool provides for thinking about risk of bias issues, in particular for reviewers who are not methodologists. They mentioned the format was clear and was helpful to tease apart various issues that contribute to the risk of bias for a study. Some mentioned they appreciate being guided to think about bias at different levels and find that helps illuminate issues within primary studies they might not have otherwise addressed.

<u>Figures</u>
One participant indicated liking the figures that RevMan produces, finding the visual component adds clarity to RoB assessments.

<u>Requires critical thinking</u>
While some participants identified the extra time involved in RoB assessments as a problem, one participant identified this as a positive aspect of the tool. This individual appreciates that the RoB tool forces authors to think critically about study methods, as opposed to merely ticking boxes as with other assessment tools.

Identified problems with the RoB tool

<u>Difficult bias domains</u>
Some participants identified that all domains within the RoB tool are challenging for authors; however, most participants agreed that the most difficult domains are selective outcome reporting, incomplete outcome data and other biases. Specific comments for each domain follow.

Allocation concealment
Few problems were identified with this domain. One participant described a scenario in which her research team could not reach consensus about the adequacy of allocation concealment for a study that used computerized randomization. Some felt appropriate allocation concealment was very likely due to the computerized approach and therefore the study is at a low risk of bias; but, others felt because a method of allocation concealment was not specifically reported the study should receive an unclear rating. The subjectivity of the assessment is therefore called into question. Another issue is the common mix-up between allocation concealment and blinding.

Sequence generation
One participant voiced a concern with the sequence generation domain that seems to result from authors not adequately understanding RoB guidance, as opposed to the guidance being unclear. This Managing Editor described that some review authors assess all studies reported to be randomized controlled trials to have adequate sequence generation, without making an appropriate assessment of the randomization method.

Blinding
There is variation in how assessments of blinding are made across authors and review groups. Some authors lump all three areas (i.e. patients, providers and outcome assessors) into one assessment, but others separate each out and add new items within RevMan tables as appropriate. For most

participants, it is unclear what method is preferred. Further, while the Handbook asks for separate assessments by outcome, not all authors do this. Some participants indicated they do not feel detail is required for each of patient, provider and outcome assessor by outcome. Instead they feel assessment by outcome only makes sense at the outcome assessor level. In general, there was agreement that for particular topic areas if patients and providers were blinded for one outcome they would be blinded for all and so a separate assessment by outcome should not be necessary. Finally, several participants indicated a struggle to provide assessments of high risk of bias for interventions where it is clearly impossible to blind the patient and/or the provider. These individuals expressed a desire for a means to provide authors with a good rating if they blind the only individual they can: the outcome assessor.

There is some confusion among authors about the meaning of double and triple blind. Some participants indicated they provide positive blinding assessments if a study is described as double blind, for example.

Incomplete outcome data

Incomplete outcome data was identified as one of the most problematic domains for both authors and Managing Editors who need to support authors in assessing this domain. While all participants agreed this domain should be included in the RoB tool, some specific concerns were raised as outlined below:

- One participant feels some concepts should be separated in order to make an appropriate assessment. For example, whether a drop out rate is described and whether it is acceptable are two different concerns; however, they are combined in the current RoB tool.
- One participant identified problems guiding authors to make a judgment regarding whether an ITT analysis had been completed. The issue is whether authors could or should assume that an ITT analysis had been completed if no drop outs or withdrawals are reported, or whether an ITT analysis needs to be reported in the Methods section.
- One participant questioned whether it was appropriate for review authors to reanalyze primary study results by imputing missing values to recreate an ITT analysis. Specifically, their concern was what such re-analyses would mean for RoB assessments.
- Several participants raised the issue of what constitutes “complete” outcome data and feel the need for a threshold. At least one review group has developed specific guidance on this issue, as RoB guidance is silent. After consulting with a statistician, this review group recommends a minimum of 80% of relevant outcome data be reported for a study to be assessed as at low risk of bias.
- One participant mentioned getting confused between incomplete outcome assessment and selective outcome reporting, and therefore suggests a different name for one or both domains.
- One participant suggests ITT should be a focus more specifically within the tool, as opposed to lumping many components into this one domain (e.g. whether everyone was randomized, everyone who was randomized was analyzed and then a differential drop out rate).
- Some expressed a lack of clarity regarding how to make assessments of missing data at different follow up periods, specifically when reasons for different rates of missing data apply for different time periods.

Selective reporting

Selective outcome reporting was identified as a particularly problematic domain. Some participants felt this domain was not helpful for making RoB assessments because proper assessments cannot be made without access to a protocol for the primary study, which is not typically the case. A few participants identified that they provide a blanket ‘unclear’ rating when a protocol is not available. Others identified uncertainty in making judgments of whether authors have measured expected outcomes for a given

topic area, because in their experience contacting study authors suggests a surprisingly large proportion of authors have not collected standard outcome data. The problem is deciphering between outcomes that are measured and not reported and outcomes that were not measured but should have been. In general, there is variation in how people interpret the guidance and assess this domain due to a lack of clarity regarding whether the protocol is needed in order to make a complete assessment or if it would suffice to just use the paper if that is all that is available.

Further, one participant was unclear whether outcome definitions should also be considered within this domain. For example, she has encountered several situations where authors have reported outcomes based on definitions or data formats that are not in standard practice.

Another issue with assessments in this domain concerns the many different ways that certain count data can be reported, with different formats resulting in different outcome interpretations. Falls were provided as one example, with falls being variously reported as number of falls, number of people who fell more than once, number who had multiple falls, average number of falls for people who fell more than once, among other ways. In such situations, reviewers express difficulty in making assessments of whether study authors are reporting their data in a way that makes their data look more positive or if what is reported is at a low risk of bias.

Other bias
Several specific concerns were raised regarding the other bias domain. Some participants feel the other bias domain should be discarded, in favor of a few more specific bias domains. Others feel the domain should remain included, but problems might instead reflect a lack of training and guidance in using the domain appropriately. A few participants feel suggestions as to what might possibly fall under this domain would be helpful for authors who feel this domain is very vague and are unsure how to provide assessments.

One specific concern is that some authors seem to use the other domain as a means to discredit a study they are unhappy with for unrelated reasons, in a sense “inventing” other types of bias to justify a poor RoB assessment.

Some participants described innovative ways they worked to overcome perceived weaknesses in this domain. At least one review group insists that review authors specify at the protocol stage what they view as being other sources of potential bias. If during the review other types of bias are identified that were not pre-specified, this review group recommends a post-hoc analysis with sufficient explanation and justification. Several other participants independently made similar suggestions as a means to improve the reliability of assessments within this domain. In this case, authors would be encouraged to specify upfront what circumstances they would perceive to result in a high risk of bias for a particular “other” domain. Alternatively, review groups might outline a pre-defined set of potential other biases specific to their content area. If authors had further suggestions for other types of biases, the review group could be open to vetting their suggestions. One participant made the suggestion that Cochrane provide guidance on how to make assessments or incorporate 'other' bias assessments into a review methodology that are determined post-hoc and not a priori at the protocol stage.

Finally, some participants identified routinely adding specific items (e.g., power calculations, funding source) to the other domain. In some cases, these items were discussed during development of the RoB tool and an explicit decision was made to exclude those items from the tool.

Conducting RoB Assessments

Experienced assessor required
One participant expressed that appropriate RoB assessments can only be made by individuals with strong methodological training, or extensive prior experience conducting methodological assessments.

Reliability
The question of reliability of RoB assessments was raised by several participants. It was recognized that in many cases a lot of discussion is needed before consistent assessments can be made, which calls into question how often these discussions take place in the context of reviews and therefore how reliable RoB data is overall. One reviewer who consistently incorporates pilot testing and the development of decision rules into his review commented that a drawback of using the RoB tool is the amount of time this process takes, especially if there are a lot of outcomes.

One participant suggested that Cochrane provide guidance regarding pilot testing RoB assessments and what that process could look like. Another reviewer suggests that RevMan be adapted to allow for multiple reviewer assessments and an inter-rater reliability calculation.

Time consuming
Several participants identified that making RoB assessments and incorporating assessments into reviews is a very time consuming process. One participant admitted this inhibits appropriate and in-depth assessments on her part. Another felt this might further discourage potential authors to engage in the already complicated Cochrane process.

Using assessments in reviews
Most participants expressed concern regarding the manner in which RoB assessments are used within systematic reviews and a lack of guidance and training materials regarding how to do this. Specifically, this is a concern of the participating Managing Editors, who are challenged to persuade their authors to reflect on what their RoB assessments mean in the context of the results of included trials. Similarly they feel challenged to persuade authors to conduct sensitivity analyses with and without studies at high risk of bias. Authors are beginning to routinely conduct RoB assessments, but the challenge lies in incorporating these assessments into reviews in a meaningful way. The majority of participants expressed a need for guidance regarding how to best summarize and incorporate RoB assessments into their reviews.

There appears to be wide variation in how review groups implement, or recommend that authors implement, RoB assessments in their reviews. In practice, some authors complete the full RoB assessment but only discuss allocation concealment in their discussion or interpretation of results, while others discuss global assessments only. Some authors do not conduct sensitivity analyses, some do for all outcomes and all domains, and still others conduct sensitivity analyses for only some domains and some outcomes. Some participants argued that their content area precludes low risk of bias assessments for particular domains (e.g., blinding) and therefore they feel compelled to include all studies in their primary analyses, for otherwise there would be a limited number of studies to include. Although some participants were unclear, it does not seem that of any of the represented review groups advise authors to restrict primary analyses to studies at low risk of bias.

Further, there appears to be a lack of clarity regarding how to make a risk of bias assessment at the study level. For example, some participants question which or how many domains are required to be

assessed as low risk of bias to make a low risk of bias assessment at the study level. A few participants raised the issue of counting domains, and suggested that inevitably some form of counting is likely to occur to make an overall assessment, even though that might be contrary to RoB guidance. As another example, some participants expressed that in some cases bias can overestimate treatment effects, but in others underestimate it, and so it is difficult to make an overall assessment as to what direction the bias is working in and at what level.

One participant identified an issue with summarizing assessments across outcomes when multiple outcome measures are used to measure the same construct (e.g., three measures used to assess quality of life). Some outcome measures have stronger validity and others less, and it is an issue for authors to make an overall judgment about risk of bias and incorporate this into their analysis when assessments differ within a given outcome.

Updating

Several participants raised the issue of updating systematic reviews as being particularly problematic from the perspective of RoB assessments. Updating requires authors to go back to studies included in the prior review and often to re-extract data to meet current standards. Review authors are often not willing to do so, and review groups are not adequately resourced to conduct the conversion to RoB on behalf of authors.

Many participants mentioned a desire for standardized Cochrane guidance regarding whether to conduct RoB assessments for old studies included in an updated review, or just the new ones. Each review group seems to have a different approach.

Variation between review groups

Variation between review group in regards to how RoB assessments are made or presented were noted in several areas. For example:

- One review group has revised the RoB tool to include several other domains (e.g., whether groups were similar at baseline, (co)-interventions, compliance, timing of outcomes) and also one question for authors to indicate whether they feel there is a fatal flaw within the primary study.
- Some review groups have made the decision to include only the first RoB figure, which is a summary by first author of each of the included studies, but not to use the second figure at all. The main issue seems to be that the graphs do not reflect the size of the studies they are summarizing, while review groups felt study size could be incorporated somehow.
- Some review groups offer their own guidance on RoB, for example by means of a summary sheet or a template to provide what they see as more practical instruction on how to develop RoB tables.
- How to interpret the guidance around the selective outcome reporting domain and whether a protocol is necessary or whether a check whether the paper agrees with itself is sufficient. At least one review group suggests that authors do not assess this domain because they do not want their reviewers to contact study authors for the protocols that they feel are necessary to make an informed assessment.
- Developing and implementing a pilot testing process and decision rules for specific assessments.
- What outcome domains should receive the greatest importance during the analysis and when otherwise incorporating results into the review.
- Whether to conduct RoB assessments for new studies only or for all studies in an update of a review.

- Adding particular types of bias to the 'other' domain on a consistent basis to address issues specific to their topic.

Some participating Managing Editors felt they wanted guidance from some central resource regarding how to guide their authors to make RoB assessments, or to have some central resource check the authority of the guidance they are offering.

Multiple study designs
While most participants recognize that RoB was not developed for non-randomized studies, many mentioned a need for Cochrane guidance on how to assess risk of bias for different study designs, for example interrupted time series, controlled before and after studies and quasi-randomized studies. Some authors are modifying RoB to meet their needs and are unclear if this practice is appropriate.

<u>Other problems</u>

Figures
Some review groups have made the decision to include only the first RoB figure in their reviews, which is a summary by first author of each of the included studies, but not to use the second figure at all. The main issue seems to be that the graphs do not reflect the size of the studies they are summarizing, while review groups felt study size could be incorporated somehow.

In addition, some participants took issue with the figures that summarize risk of bias at the study level, where throughout the review assessments are encouraged at the outcome level. These participants felt figures at the outcome level would be more appropriate.

One participant suggested to use black, white and grey instead of red, yellow and green in the figures, as the basis for the current colour choice is unclear.

Wording
Several individuals indicated they find the yes and no wording complicated and consequently often mix the meaning between high and low risk of bias.

A few participants also indicated they would prefer to also have a 'medium' or 'moderate' category in addition to an unclear, because at times they feel either a high or a low assessment is inappropriate based on reported methods. For example, in some situations it is clear what the authors did; but the study methods do not warrant a strong assessment of either high or low risk of bias. One participant indicated they currently use the unclear category as a middle category between high and low risk.

Guidance materials

Feedback was provided regarding the Handbook chapter, workshops, online training and guidance materials in general. Overall, most felt the guidance is clear; however, there is a challenge in persuading authors to follow the recent and more complicated guidance and truly understand what it means. Generally, most participants agreed that more training needs to be available.

<u>Handbook</u>

Most participants identified using the handbook as guidance for specific issues, but few read the handbook from start to finish. Many do not feel it is reasonable to expect authors to read the very detailed guidance in the handbook due to its complexity.

<u>Workshops</u>
For many participants, hands-on workshops are a preferred method of training, as open discussions can occur about problematic issues. Several, however, recognize that workshops can be difficult to organize and attend and therefore participation is often low. A need was expressed for various levels of RoB workshops, beyond the introductory workshops typically offered at symposia and colloquia. Many feel the concepts are fairly sophisticated and take discussion and practice to grasp. Three levels (i.e. introductory, intermediate and advanced) were suggested. Further, many argued for more than one day workshops, feeling that more time is needed to adequately understand each risk of bias domain plus to ensure sufficient time for hands-on work and discussion regarding how to use assessments in a review.

A few participants expressed that the RoB training available within the standard author training is insufficient. They felt this to be a good venue to introduce the concepts, the RoB tool and available guidance, but that other more in-depth training is needed to explain the specifics.

<u>Online training</u>
The concept of online training was raised several times. Managing Editors felt online training would be helpful to refer to review authors, especially when it is clear they have not (and will not) read the Handbook. Most feel online training is a more financially sound option over in-person workshops when several reviewers need to be trained.

The concept of webinars (real-time web-based seminars) was also raised as a potential training tool.

<u>Guidance materials in general</u>
Several suggestions were made as to how to augment existing training materials. Some examples of areas that participants felt required further guidance follow:

- How to make an assessment of whether an ITT analysis has been done if there are no drop outs and no withdrawals. In this situation, can the assumption be made that an ITT analysis has been done?
- How to report methods related to risk of bias within study protocols and reports, for example regarding how to group included studies within meta-analyses and how to summarize RoB assessments.
- How to incorporate RoB assessments into reviews (i.e. analyses, results, conclusions, plain language summaries)
- How to summarize RoB assessements from the outcome, study and review levels
- To follow PRISMA (Preferred Reporting Items for Systematic reviews and Meta-Analyses) guidelines when preparing review reports
- History of how the collaboration moved from the prior practice of quality assessment to the current model of risk of bias assessments, including the rationale so that authors familiar with the old way of doing things have a basis to move forward.
- How to make judgments when information is poorly reported. In some cases, it is possible to make a best guess, but in some situations strong arguments can also be made for other ratings because the information is not comprehensively reported.

- How to make assessments when trials report being double-blinded, specifically how to determine what ‘double-blind’ means and how different situations translate into different potentials for risk of bias.
- How to assess studies that include outcomes and/or interventions that cannot be blinded to each of participant, provider and outcome assessor. Many participants feel such studies should not be marked as high risk of bias. In most reviews with this issue, all studies will be assessed at a high risk of bias and so guidance regarding how to otherwise appropriately distinguish risk of bias between included studies would be helpful. Many feel a need to somehow highlight studies with better and worse methodological approaches within such groups.
- Several times concerns were raised regarding bias domains not included in the tool that participants felt should be, for example sample size calculations and funding. For many of these domains, the focus group facilitator described that the issue was discussed during the tool development process and an explicit decision was made to exclude the item for a particular reason. In these cases it seems helpful to include a history within the guidance materials to outline the decision and reasoning; because in many cases review authors are adding in these domains within the 'other' category and therefore unknowingly going against Cochrane policy.

The examples provided within existing guidance materials received a lot of discussion. Generally, the examples are very well liked by focus group participants, as they find them helpful to make decisions regarding specific assessments. Several participants requested further examples, however, and provided specific feedback as follows:

- While several examples are provided, even more would be helpful to clarify a wider range of situations
- Review group specific examples might be warranted that could be verified by methods groups in advance
- Examples based on complex interventions and/or psychosocial interventions are requested due to a lack of clarity regarding RoB assessments in these areas
- An electronic repository of examples and corresponding assessments might be helpful
- An issue was raised with a particular existing example under the randomization domain that lists a description as 'probably done because they did it in the previous trial'. This example is seen to raise issues with some authors who know authors of primary studies they are reviewing and who automatically give positive assessments to these authors following this example.
- Certain authors use the examples as a ‘pick list’ of options from which to make their assessments without truly understanding the issues. It might be necessary to indicate that the examples are just that and a real understanding of bias-related issues is required before making an assessment.

<u>Other discussion points</u>

- The concept of a network of individuals to discuss issues with RoB assessments, while they are being made in the context of a review, was raised a few times. The network could include review authors with experience and/or methods group representatives and have the goal to have a knowledgeable person available to ask specific questions to when in the process of conducting a review.
- Some recognize the Handbook chapter as very useful but feel that a summary document is also needed. To this end, some review groups have developed their own summary documents. One participant offered a suggestion for a CONSORT- or PRISMA-like explanation and elaboration document for each item within the risk of bias tool.

- A training package for people who need to train their staff to use the tool was described as potentially helpful.
- Some identified a need for RevMan training relating to building RoB tables, although they were unclear whether this exists already in the RevMan manual. Further, a desire for some guidance within RevMan regarding how to use RoB assessments in subsequent parts of the review was identified as likely helpful for authors who do not read the Handbook.
